# A universal scaling law of intra-urban inequality

**DOI:** 10.1038/s41467-026-73015-1

**Published:** 2026-05-09

**Authors:** Conghong Huang, Xiaodan Liu, Shibin Zhang, Zongyang Jin, Nan Xu, Weixin Ou

**Affiliations:** 1 College of Land Management, Nanjing Agricultural University, Nanjing, China; 2 National & Local Joint Engineering, Research Center for Rural Land Resources Use and Consolidation, Nanjing, China; 3 Key Laboratory for Geo-Environmental Monitoring of Great Bay Area, Ministry of Natural Resources, Shenzhen University, Shenzhen, China; 4 Guangdong Key Laboratory of Urban Informatics, Shenzhen University, Shenzhen, China; 5 School of Architecture and Urban Planning, Shenzhen University, Shenzhen, China

**Keywords:** Environmental impact, Sustainability

## Abstract

Large-scale urban agglomeration yields economic benefits but often coincides with pronounced environmental and social disparities. While aggregate urban attributes follow predictable scaling laws, how intra-urban inequality scales with city size remains unclear. To bridge this gap, we analyze over 11,000 global urban centers using multi-source satellite data to quantify inequalities in thermal exposure, green space, and economic activity. Here we show that all three dimensions follow super-linear scaling laws with city population size. Specifically, a doubling of city population is associated with an approximately 8-9% higher inequality Gini coefficient. Furthermore, these scaling relationships are significantly modulated by national socioeconomic development and background climate: green space and economic inequalities exhibit steeper scaling in low-income nations, whereas thermal inequality scales most acutely in arid climates. Consequently, achieving equitable urban development requires targeted interventions rather than relying on passive scale effects.

## Abstract (web summary)

Intra-urban inequality scales super-linearly with city size. Analyzing over 11,000 cities globally, this study reveals that a doubling in population exacerbates spatial disparities in thermal, green space, and economic exposure by 8-9%.

## Introduction

Cities are the primary arenas where humanity experiences the impacts of global change^1,2^. The unprecedented scale and pace of global urbanization, particularly over the past several decades, has fundamentally transformed the planet’s land surface and poses significant challenges for sustainability^3^. Fueled by rapid global urbanization^4^, cities increasingly function as crucial nodes for economic dynamism and technological advancement. However, this progress is often accompanied by profound social and environmental costs. A critical but often overlooked factor is the spatial footprint of urbanization: while higher urban density drives economic agglomeration, it may inadvertently intensify local environmental stressors. For instance, densification presents significant challenges for preserving green spaces and maintaining microclimatic comfort^5^, creating a local health paradox where the benefits of urban growth are unevenly distributed^6^.

To understand these trade-offs, we focus on three critical dimensions of the urban environment that are directly linked to human health and wellbeing: thermal exposure, green infrastructure, and economic activity. First, land surface temperature (LST) serves as a key indicator of heat exposure risk. The uneven distribution of urban heat often creates a landscape of thermal inequity, where marginalized populations are disproportionately exposed to heat stress^7–9^. Second, urban green space (UGS) acts as a vital health resource. Systematic reviews confirm that access to green space significantly reduces all-cause mortality by mitigating heat islands, encouraging physical activity, and facilitating psychological restoration^10,11^. Third, nighttime light (NTL) intensity provides a robust high-resolution proxy for the intensity of economic activity at the micro-scale^12,13^, reflecting the spatial distribution of economic wellbeing. Consequently, understanding the fundamental principles that govern urban organization and its socio-ecological consequences is a critical scientific challenge for achieving sustainable urban development^1,14^.

A promising framework for uncovering these principles is urban scaling theory, which has demonstrated that many aggregate urban attributes, such as infrastructure and economic output, and social dynamics, follow predictable, power-law scaling relationships with city population size^14–17^. Typically, socioeconomic outputs like wealth and innovation scale super-linearly (exponent > 1), meaning they grow faster than the population (suggesting increasing returns), while infrastructure scales sub-linearly, indicating economies of scale (i.e., growing slower than population, resulting in material savings)^14^. These scaling laws have been shown to be remarkably consistent across different nations and historical periods. However, while scaling laws are powerful tools for characterizing the macro-structural state of urban systems^17^, they are primarily based on cross-sectional data and should be distinguished from the longitudinal trajectories of individual cities, which may diverge due to path dependencies^18,19^. Furthermore, this aggregate-level perspective, which often treats cities as zero-dimensional objects in a one-value-per-city analysis, largely overlooks a crucial dimension of urban systems: the stark inequalities and spatial heterogeneities that exist within cities^17,20^. Indeed, the challenge of enduring neighborhood inequality is increasingly recognized as a fundamental barrier to achieving urban sustainability^21^.

Parallel to the development of scaling theory, a substantial body of environmental justice research has documented the inequitable distribution of environmental amenities and risks within urban areas. Studies have consistently shown that low-income and minority communities are disproportionately exposed to higher levels of urban heat^7,22–25^ and have less access to cooling infrastructure, such as green spaces^26^. This phenomenon, where desirable green amenities are often concentrated in wealthier neighborhoods, is sometimes termed the luxury effect^27,28^. While these studies effectively demonstrate the existence of environmental inequality, they often rely on case studies of individual or small numbers of cities^29^. A unifying, predictive framework that connects the emergence of these inequalities to the fundamental process of urban growth across the globe remains a critical research gap, a point increasingly recognized within the urban scaling community itself^17^. Recent work has begun to bridge this divide, suggesting that macroscopic scaling laws may in fact arise from the heavy-tailed, unequal distributions of productivity at the micro-level^20^, but it is unknown if a similar principle governs the scaling of environmental and economic inequality itself. This omission not only limits the theoretical scope of urban scaling but also hinders the development of a predictive, science-based approach to achieving urban equity.

**Fig. 1:**
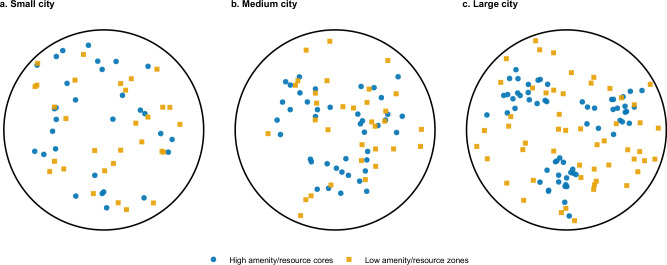
Conceptual framework for the scaling of intra-urban inequality. The schematic illustrates the hypothesis that inequality scales super-linearly with city size due to spatial sorting. In small cities (**a**), high-amenity resources (blue circles) and low-amenity zones (red squares) are relatively mixed, exhibiting a stochastic distribution. In medium-sized cities (**b**), spatial sorting is discernible, with resources showing partial aggregation. In large cities (**c**), high amenities are concentrated into distinct clusters or pockets, which are spatially segregated from lower-amenity areas. The observation that larger cities exhibit a more clustered spatial structure compared to the mixed distribution in smaller cities underlies the disproportionate increase in inequality (Gini coefficient) relative to population size (*α* > 0). Note: The spatial separation depicted is a schematic abstraction to illustrate the statistical divergence of inequality (widening gap between high-amenity and low-amenity areas) and does not represent a specific geographic layout (e.g., East-West divide).

Here, we address this critical gap by proposing and testing the hypothesis that intra-urban inequality itself follows predictable, super-linear scaling laws with city size (Fig. 1). To do this, we construct a comprehensive global dataset for over 11,000 urban centers to: (1) quantify the global scaling relationship between thermal (LST), green space, and economic (NTL) inequalities, and city population; and (2) determine how these scaling laws are modulated by national socioeconomic development and background climate. Our results reveal a consistent super-linear scaling for all three inequality types, indicating that inequality is systematically higher in larger urban centers. We further validate that population density remains a robust proxy for urban form compared to geometric indices (Supplementary Fig. 5). This research contributes to advancing the understanding of urban environmental justice from case-specific description toward a more predictive, theory-based approach, providing a quantitative foundation to inform policies aimed at achieving equitable and sustainable urbanization globally.

## Results

### Global scaling laws of urban inequality

**Fig. 2:**
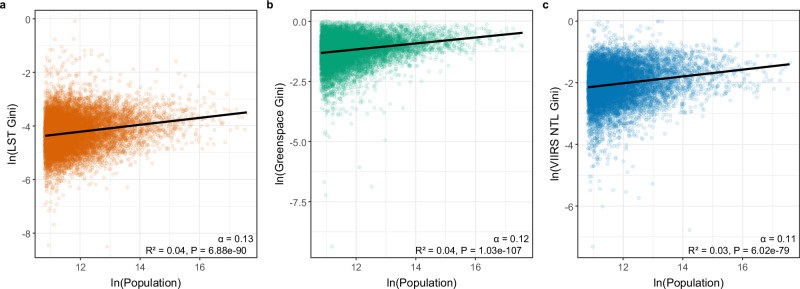
The global scaling laws of urban inequality. Super-linear scaling of intra-urban inequality with city population for *n* = 11,210 independent urban centers. Relationships are shown on a log-log scale for **a** thermal inequality (Land Surface Temperature, LST Gini), **b** green space inequality (Green Space Gini), and **c** economic inequality (Visible Infrared Imaging Radiometer Suite Nighttime Lights, VIIRS NTL Gini). Each point represents a city. The solid black line is the ordinary least squares regression fit. The scaling exponent (*α*), coefficient of determination (*R*^*2*^), and exact P-value are reported for each model within the plot panels. *P*-values were derived from two-sided *t*-tests on the regression slopes without adjustments for multiple comparisons.

Our analysis, encompassing over 11,000 cities worldwide (based on data circa 2024, defined as high-density urban centers, see Methods) (Supplementary Fig. 1), reveals that intra-urban inequalities are not random but follow predictable scaling laws with city size. While population size serves as the primary predictor of the global scaling trend, we anticipate that its explanatory power for the variance in inequality will be modest, given the substantial cultural, economic, and morphological diversity of cities worldwide that creates significant local deviations from the universal law. We find that for all three types of inequality examined, which are thermal, green space, and economic, inequality systematically increases with the size of the urban population. As shown in Fig. 2, a robust super-linear relationship emerges when plotting the Gini coefficient against city population on a log-log scale. Specifically, for thermal inequality (LST Gini), the scaling exponent (*α*) is 0.13 (*R*^*2*^ = 0.04, *P* < 0.001), indicating that a doubling of a city’s population is associated with a (2^0.13^ − 1), or approximately a 9% increase in thermal inequality (Fig. 2a). A similar super-linear scaling is observed for green space inequality (Green Space Gini), with an exponent of *α* = 0.12 (*R*^*2*^ = 0.04, *P* < 0.001), indicating that a doubling of city population is associated with a (2^0.12^ − 1), or approximately a 9% increase in green space inequality (Fig. 2b). Economic inequality, proxied by Visible Infrared Imaging Radiometer Suite (VIIRS) NTLs Gini, also follows this pattern, scaling with an exponent of *α* = 0.11 (*R*^*2*^ = 0.03, *P* < 0.001), which corresponds to an approximately 8% increase for every doubling of city population (2^0.11^ − 1) (Fig. 2c). While the proportion of variance explained by population size alone is modest, the high statistical significance across thousands of cities points to a consistent pattern of urban organization. Larger cities tend to be inherently more unequal in their distribution of environmental amenities and economic activity.

### Socioeconomic and climatic modulation of inequality scaling

**Fig. 3:**
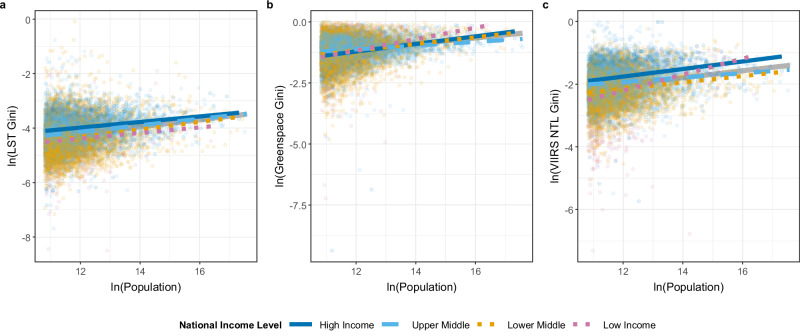
Socioeconomic modulation of urban inequality scaling laws. Scaling relationships between intra-urban inequality and population size, stratified by four national income levels (High, Upper Middle, Lower Middle, and Low Income). Panels show results for **a** thermal inequality, **b** green space inequality, and **c** economic inequality. Each semi-transparent point represents an individual city, colored according to its income level using a colorblind-safe palette. The colored lines, styled with different line types for each group, show the ordinary least squares (OLS) regression fit, while the thick light gray line in the background represents the global average trend for reference. Data represent *n* = 11,210 independent urban centers.

While the super-linear scaling of inequality is a global phenomenon, we find that its intensity is profoundly modulated by both national socioeconomic conditions and background climate. The analysis stratified by national income level (see Methods for income classification details) reveals significant divergence in the scaling of both green space and economic inequality (Fig. 3). For green space inequality (Fig. 3b), the scaling exponent for low-income countries (*α* = 0.24) is substantially and significantly steeper than for upper-middle-income countries (*α* = 0.08, interaction *P* < 0.001). This suggests that cities in lower-income nations experience a much more rapid exacerbation of green space in larger cities. A similar pattern is observed for economic inequality (Fig. 3c), where the scaling exponent in low-income countries (*α* = 0.26) is significantly greater than in upper-middle-income (*α* = 0.07, interaction *P* < 0.001) and high-income countries (*α* = 0.12, interaction *P* < 0.001). In contrast, the scaling of thermal inequality appears less sensitive to national income levels. Only the lower-middle-income group shows a significantly different slope from the high-income baseline (interaction *P* = 0.035), suggesting a more universal physical process underlying thermal disparities (Fig. 3a).

**Fig. 4:**
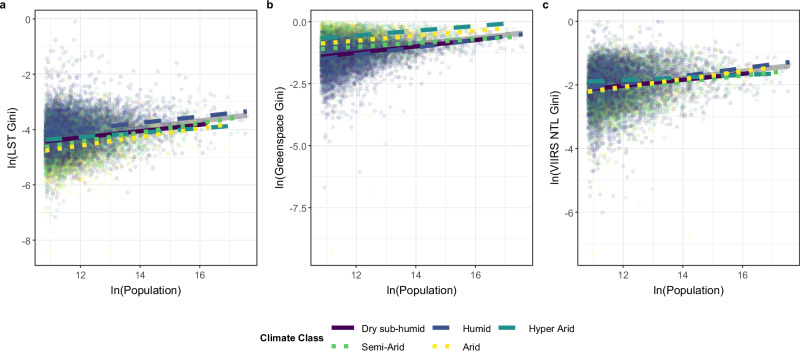
Climatic modulation of urban inequality scaling laws. Scaling relationships between intra-urban inequality and population size, stratified by five major climate classes. Panels show results for **a** thermal inequality, **b** green space inequality, and **c** economic inequality. Each semi-transparent point represents an individual city, colored according to its climate class using a colorblind-safe palette. The colored lines show the ordinary least squares (OLS) regression fit for each class, while the thick light gray line in the background represents the global average trend for reference. Data represent *n* = 11,210 independent urban centers.

Background climate also modulates these scaling laws (see Methods for climate classification details), albeit in a more nuanced manner than socioeconomic conditions (Fig. 4). The scaling of thermal inequality (LST Gini) shows the most pronounced climatic influence. The scaling exponent is steepest in semi-arid (*α* = 0.17) and arid (*α* = 0.16) climates and is significantly flatter in humid climates (*α* = 0.12, interaction *P* = 0.049). This finding suggests that the physical constraints of the environment, likely related to water availability for evaporative cooling, play a key role in shaping thermal disparities as cities increase in size (Fig. 4a). Contrary to our initial global-level analysis, we find that the scaling of green space inequality also exhibits a significant climatic modulation. Specifically, the scaling exponent in humid climates (*α* = 0.15) is significantly steeper than in arid climates (*α* = 0.10, interaction *P* = 0.006), indicating a more rapid exacerbation of green space inequity in wetter regions once a city reaches a certain size (Fig. 4b). The scaling of economic inequality shows the weakest climatic influence, with only hyper-arid cities showing a significantly flatter scaling exponent compared to arid cities (interaction *P* = 0.037), suggesting its spatial distribution is more strongly governed by socioeconomic drivers (Fig. 4c).

### Synthesis and robustness of findings

**Fig. 5:**
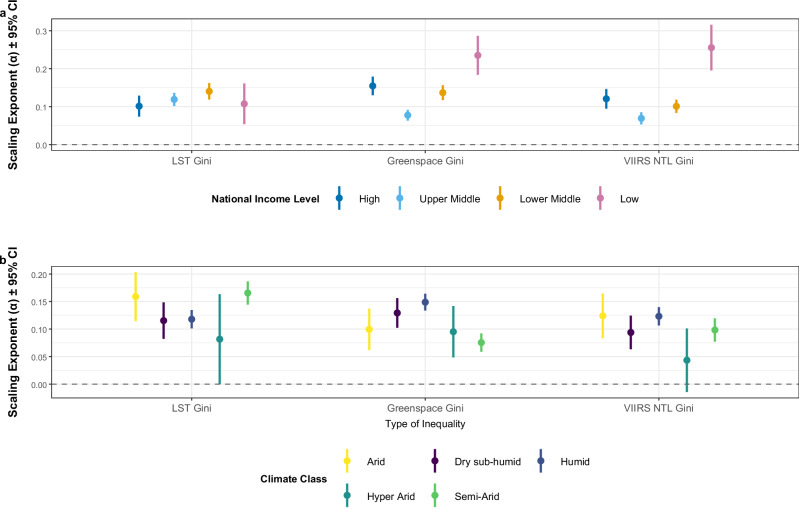
Synthesis of scaling exponents (*α*) for urban inequality. A quantitative comparison of scaling exponents (*α*) reveals the differential impacts of socioeconomic and climatic factors. Data are presented as the scaling exponent (*α*) ± 95% Confidence Intervals (error bars). The points represent the estimated scaling exponents from stratified linear regressions. A value of *α* > 0 signifies super-linear scaling (inequality worsens with city size). **a** Scaling exponents stratified by national income level. **b** Scaling exponents stratified by major climate zones. The exact sample sizes (*n*) for the subgroups are: High income (1550), Upper Middle (4143), Lower Middle (4569), Low income (948); Humid (5458), Semi-Arid (2909), Dry sub-humid (1466), Arid (1094), and Hyper Arid (283) independent urban centers. Statistical significance was assessed using two-sided *t*-tests on *t*he regression coefficients.

A direct comparison of the scaling exponents quantifies these modulating effects (Fig. 5). The starkest contrasts are visible in green space and economic inequality, where the scaling exponents for low-income countries are more than double those of upper-middle and high-income countries. The non-overlapping 95% confidence intervals confirm the statistical significance of these differences.

Our findings are robust to methodological choices (detailed in Methods: Calculation of Inequality Metrics). A quantile regression analysis reveals that the super-linear scaling relationship holds not only for the median city but also for the most equal (10th percentile) and most unequal (90th percentile) cities. This confirms the universality of the scaling law across different levels of inequality (Supplementary Fig. 2). For green space inequality, the scaling exponent is notably larger for more equal cities (*α* = 0.21 at 10th percentile) than for already unequal cities (*α* = 0.05 at 90th percentile), suggesting a potential saturation effect in highly unequal urban systems. Furthermore, our results are not sensitive to the specific spatial radius used to calculate the Gini coefficients (Supplementary Fig. 3).

In an in-depth study of the mechanism, a residual analysis shows that after controlling for population size, urban form, proxied by population density, explains a significant portion of the remaining variance in inequality (Supplementary Fig. 4). To validate the robustness of this proxy, we further conducted a sensitivity analysis using three geometric indices (compactness, fractal dimension, and elongation) and road network density. As shown in Supplementary Fig. 5, population density consistently exhibited the strongest explanatory power for the variance in inequality residuals across all dimensions compared to geometric forms. More compact (denser) cities tend to have significantly lower thermal (*r* = −0.23, *P* < 0.001) and economic (*r* = −0.33, *P* < 0.001) inequality than their more sprawling counterparts of the same population size. Conversely, compactness is associated with significantly higher green space inequality (*r* = 0.17, *P* < 0.001), suggesting a potential trade-off between land use efficiency and equitable green space distribution in urban planning. Finally, we find a significant, albeit weak, positive correlation between economic inequality and green space inequality (*r* = 0.05, *P* < 0.001). However, we observe no significant correlation between green space inequality and thermal inequality at the global scale (*r* = 0.001, *P* = 0.89), suggesting a more complex pathway from socioeconomic status to thermal risk than a simple mediation through green space (Supplementary Fig. 6). Additionally, we observe a significant, positive correlation between thermal and economic inequality (*r* = 0.176, *P* < 0.001), suggesting that areas with higher economic activity also tend to experience greater heat exposure, potentially due to a higher concentration of impervious surfaces and anthropogenic heat sources (Supplementary Fig. 6).

## Discussion

Our global analysis of over 11,000 cities reveals a systematic feature of urbanization: intra-urban inequality itself follows a predictable, super-linear scaling law with city size. This result warrants careful interpretation. Unlike extensive urban metrics, such as GDP, for which super-linearity is typically defined by a scaling exponent greater than one (*α* > 1), for an intensive, ratio-based metric like the Gini coefficient, the baseline expectation is an absence of scaling (*α* = 0). Therefore, any statistically significant positive exponent indicates that inequality is systematically exacerbated with city size, a phenomenon defined as super-linear in this context. This finding provides a crucial bridge between two major fields of urban science. On one hand, it extends urban scaling theory, which has successfully described how aggregate city-level attributes scale with population^14,15^, by demonstrating that the internal distribution of these attributes is also a scalable property. On the other hand, it helps advance environmental justice research from predominantly case-specific descriptions of inequity^22,24^ toward a more predictive framework capable of characterizing the structural state of inequality as a function of urban scale. The modest *R*^*2*^ values in our global models do not diminish the significance of this finding; rather, they highlight that while population size provides a universal baseline for the scaling of inequality, other factors, which we explore, play a critical role in modulating this process.

A key contribution of our work is the disentanglement of how socioeconomic development and background climate differentially modulate the scaling of various inequality types. A key finding is the strong modulation of green space and economic inequality by national income level. The scaling exponents for these inequalities are substantially steeper in low-income countries compared to high- and upper-middle-income nations. This suggests that larger cities in low- and middle-income countries face a much more rapid intensification of spatial disparities in both ecological amenities and economic activity. This aligns with theories of uneven development and provides large-scale empirical evidence that the capacity to manage and distribute resources equitably scales with national wealth. The luxury effect, where green space is associated with affluence^27^, appears to be a far more potent driver of inequality in contexts of resource scarcity and rapid, often informal, urbanization. In contrast, the scaling of thermal inequality with city size showed less sensitivity to income levels but was strongly modulated by background climate. Specifically, the exacerbation of thermal inequality in larger cities is most acute in semi-arid (*α* = 0.17) and arid (*α* = 0.16) regions. This finding underscores the interplay between social processes and physical constraints. While green space distribution is a matter of planning and investment, thermal landscapes are also governed by biophysical laws where factors like water availability for evaporative cooling become critical^30^. Our results suggest that in water-limited environments, the cooling benefits of any existing green space are highly contested and likely captured by wealthier communities, thus resulting in steeper thermal inequality gradients in larger cities.

Our findings have practical implications for sustainable urban development and climate justice. Understanding the complex trade-offs between multiple environmental and socioeconomic outcomes is a central challenge for sustainability in an urbanizing planet^31^, and our observation of a consistent scaling law for inequality quantifies a key mechanism driving one such trade-off. This inherent scaling property of urban systems complicates the ambitions of the United Nation’s Sustainable Development Goals, particularly SDG 10 (Reduced Inequalities) and SDG 11 (Sustainable Cities and Communities)^1^. It suggests that, left to their own devices, larger cities have an inherent tendency to become more unequal. This structural association between urban scale and inequality implies that achieving equitable urban development cannot be a passive goal but requires proactive, targeted interventions. Our results provide a quantitative basis for such interventions. For instance, the finding that low-income nations are on a steeper trajectory of green space inequality highlights the urgent need for well-funded policies that prioritize equitable greening strategies, which require robust funding and financing mechanisms^32^. Our analysis of urban form (Supplementary Fig. 4) indicates that promoting compact urban development could be a co-benefit strategy, simultaneously mitigating the scaling of thermal and economic inequality, although potential trade-offs with green space equity must be carefully managed. Furthermore, in arid and semi-arid regions where thermal inequality scales most acutely, urban cooling must be prioritized as an essential public health imperative, necessitating policies that integrate water justice with urban planning through distributed, water-efficient cooling solutions in vulnerable neighborhoods to prevent climate resilience from becoming a privilege of wealth.

Our study has several limitations. First, our scaling analysis is based on cross-sectional data. While this approach effectively characterizes the macro-structural state of the global urban system^17^, it should not be conflated with the longitudinal trajectories of individual cities. As noted by Pumain^33^, Depersin and Barthelemy^18^, and Keuschnigg et al.^19^, longitudinal scaling exponents may diverge from cross-sectional ones due to path dependencies. Second, regarding urban form, we used population density as a primary proxy. Although we validated this against geometric metrics (e.g., compactness, fractal dimension) and found density to be the most robust predictor of inequality residuals (Supplementary Fig. 5), we acknowledge that density alone does not capture the full complexity of urban morphology (e.g., street canyon effects on heat trapping). Third, we use remote sensing-based proxies for complex phenomena; LST is not equivalent to human thermal comfort, and NTLs capture only a facet of economic activity. Fourth, our Gini coefficient approach quantifies overall inequality but does not reveal the specific social groups (e.g., defined by race or ethnicity) who are most affected. Finally, our scaling analysis reveals strong correlations, but establishing the precise causal mechanisms requires further, more detailed investigation, potentially integrating micro-level data as has been demonstrated by Arvidsson et al.^20^

In conclusion, our research demonstrates that intra-urban inequality follows a predictable scaling law of urban organization. By revealing that cities are structured to be more unequal in predictable ways, we provide a quantitative framework and a practical, data-driven tool for urban science and planning. The future of sustainable urbanization hinges not only on managing aggregate growth but on actively shaping the distributive patterns that emerge from it. The challenge for the coming decades will be to find development pathways that can break the scaling laws of inequality, bending the curve towards a more just and equitable urban future for all.

## Methods

### Studied cities

Our study is based on a final sample of 11,210 urban centers worldwide, with broad coverage across different socioeconomic and climatic regions (Supplementary Fig. 1). To ensure a globally consistent and comparable definition of urban areas, we utilized the Global Human Settlement Urban Center Database version R2024A (GHS-UCDB)^34,35^, which initially contained data for 11,422 locations. In this database, an Urban Center is defined as a cluster of contiguous grid cells with a density of at least 1500 inhabitants per km² and a total population of at least 50,000, representing the high-density urban core. Our final sample includes only those urban centers for which valid data were available for all variables of interest (population, LST, green space, and NTLs), excluding any entries with missing or non-positive values.

### Remote sensing and geospatial data processing

All remote sensing data for this study were acquired and processed on the Google Earth Engine (GEE) platform, a cloud-based platform for planetary-scale environmental data analysis^36^.

To derive the green space cover for each urban center, we used Sentinel-2 Level-2A surface reflectance imagery^37^, accessed via the GEE data collection, to calculate the Normalized Difference Vegetation Index (NDVI)^38^. The NDVI was computed for the 2024 growing season, which was defined as May–September for the Northern Hemisphere and November–March for the Southern Hemisphere. A threshold of NDVI > 0.39 was applied to generate a binary green space map. This threshold was determined through a rigorous validation process. We first selected Beijing as a case study and generated multiple binary green space maps using a series of NDVI thresholds ranging from 0.20 to 0.50 at 0.01 intervals. In this context, an NDVI value of 0.20 represents the lower limit of vegetation detection, typically corresponding to bare soil, impervious surfaces, or highly mixed pixels with minimal vegetative cover. Conversely, a value of 0.50 strongly indicates dense and healthy vegetative canopies. Testing this specific range (0.20 to 0.50) is critical because it precisely captures the transitional boundary required to effectively separate functional UGSs from non-vegetated urban structures. These maps were then overlaid onto high-resolution satellite images from Environmental Systems Research Institute (ESRI) within Quantum Geographic Information System (QGIS, version 3.34) for visual comparison. The threshold of 0.39 was identified as best capturing the spatial patterns and overall extent of green space visible on the high-resolution imagery. To ensure its generalizability, we subsequently applied this threshold to cities in diverse climatic regions, including Dubai, New York, and London, confirming its robust performance in accurately extracting green space patterns across different urban environments.

Summertime (defined as June–August for the Northern Hemisphere and December–February for the Southern Hemisphere) LST was computed as the average from 2022 to 2024 using Landsat 8 and 9 Collection 2, Tier 1, Level-2 science products. We specifically used the ‘ST_B10’ (thermal) band, which provides surface temperature values, from these collections. The computation was performed within GEE, following a previously published open-source code and algorithm^39^, which implements the statistical mono-window (SMW) algorithm. Specifically, the top of atmosphere (TOA) brightness temperature from the thermal band was converted to actual LST using empirical relationships parameterized by surface emissivity and total column water vapor (TCWV). To achieve this, surface emissivity was dynamically estimated by combining bare soil emissivity from the ASTER Global Emissivity Dataset (ASTER GED) with Fractional Vegetation Cover (FVC) derived from the NDVI. Atmospheric effects were corrected by utilizing TCWV data from atmospheric reanalysis models to select the appropriate SMW algorithm coefficients.

We used the median composite of the VIIRS Stray Light Corrected Nighttime Day/Night Band Composites Version 1 data^40^ for the years 2022–2024 as a proxy for the intensity of economic activity. This dataset was also sourced from the GEE data catalog.

To enable population-weighted analyses, we utilized the 100 m resolution gridded population count data from the WorldPop project, specifically the ‘Global Project Population Data’ product.

### Calculation of inequality metrics

To quantify intra-urban inequality, we calculated population-weighted Gini coefficients based on neighborhood-level exposure. This approach assesses the inequality of conditions experienced by the population rather than the raw spatial distribution of the variables themselves. The process involved a focal analysis centered on each 100 m population grid cell. For each population cell, we calculated the mean green space coverage, LST, and NTL intensity within a surrounding 500 m radius buffer. The population count of the central grid cell was then paired with these neighborhood-level mean values. This resulted in a series of paired (population, exposure value) data points for each city. The Gini coefficient, a measure of statistical dispersion ranging from 0 (perfect equality) to 1 (perfect inequality), was then computed for each city and each variable. This calculation was performed in the R programming environment (version 4.5.1)^41^ using the gini.wtd function from the dineq package, with the neighborhood-level mean values serving as the value vector and the corresponding cell populations as the weight vector. The primary results presented in this paper are based on the 500 m buffer analysis, while additional calculations using 300 and 800 m buffers were performed for sensitivity analysis (Supplementary Fig. 3).

### Urban form and infrastructure metrics

To characterize urban form beyond simple population density, we calculated three geometric indices for each urban center based on the GHS-UCDB vector boundaries: (1) Compactness Index (CI), calculated as the Isoperimetric Quotient (CI = 4π *A* / *P*^2^), where *A* is the area and *P* is the perimeter. This index ranges from 0 to 1, with values closer to 1 indicating a more compact, circular shape. (2) Fractal Dimension (D), estimated using the perimeter-area relationship approximation (*D* = 2 ln *P* / ln *A*), representing the complexity of the urban boundary^15^. (3) Elongation Ratio, defined as the ratio of the length of the major axis to the minor axis of the bounding box enclosing the urban center. Additionally, Road Network Density was included as an infrastructure-based morphological metric, derived from the GHS-UCDB data (‘IN_ROA_DEN’ variable). These metrics were used in a sensitivity analysis to validate the robustness of population density as a proxy for urban form.

### Scaling law analysis

To test our primary hypothesis that urban inequality scales with city size, we employed scaling analysis, a framework established to describe how urban metrics change with population size^14,15^. We performed Ordinary Least Squares (OLS) regression on the log-transformed variables. The model is specified as:

1 $${{\mathrm{ln}}}(G)=\alpha {{\mathrm{ln}}}(N)+\beta$$

where G is the Gini coefficient for a given inequality type, N is the total population of the urban center from the GHS-UCDB database, *α* is the scaling exponent, and β is the intercept. An exponent *α* > 0 indicates super-linear scaling, where inequality increases disproportionately with city size.

To investigate the modulation effects, we stratified the global sample by socioeconomic and climatic factors. The socioeconomic stratification was based on the four national income levels provided by the GHS-UCDB, which aligns with the World Bank classification (fiscal year 2024) based on 2022 GNI per capita: Low Income (<= $1135), Lower-Middle Income ($1136–$4465), Upper-Middle Income ($4466–$13,845), and High Income (> $13,845)^42^. The climatic stratification was based on the Aridity Index (AI) from the Global-AI_ET0_v3 dataset^43^, which was classified into five major climate types (Hyper Arid, Arid, Semi-Arid, Dry sub-humid, and Humid) according to the United Nations Environment Program (UNEP) standard^43^: Hyper Arid (AI < 0.03), Arid (0.03 ≤ AI < 0.20), Semi-Arid (0.20 ≤ AI < 0.50), Dry sub-humid (0.50 ≤ AI < 0.65), and Humid (AI ≥ 0.65). To statistically test whether the scaling exponents (*α*) were significantly different between groups, we used an extension of Eq. 1 that included an interaction term between the log-transformed population and the categorical moderator variable (e.g., income level). All statistical analyses were conducted in R version 4.5.1^41^.

### Reporting summary

Further information on research design is available in the Nature Portfolio Reporting Summary linked to this article.

## Supplementary information

Supplementary Information

Reporting Summary

Transparent Peer Review file

## Data Availability

The third-party datasets used in this study are publicly available. The Global Human Settlement Urban Center Database (GHS-UCDB) is available from the European Commission’s Joint Research Center. Satellite imagery from Sentinel-2 (GEE ID: COPERNICUS/S2_SR_HARMONIZED), thermal data from Landsat 8/9 (GEE ID: LANDSAT/LC08/C02/T1_L2, and LANDSAT/LC09/C02/T1_L2), and the VIIRS NTLs data (GEE ID: NOAA/VIIRS/DNB/MONTHLY_V1/VCMSLCFG) were sourced from the GEE data catalog. These datasets are provided by the European Space Agency, the U.S. Geological Survey, and the Earth Observation Group at the Payne Institute for Public Policy, respectively. The WorldPop population data are available at worldpop.org and GEE (GEE ID: WORLDPOP/GP/100 m/pop). The processed, city-level dataset generated in this study, which includes the source data underlying all main and Supplementary Figs. (provided as dataMerged.csv and dataMerged.rds), has been deposited in the Figshare database under accession code 10.6084/m9.figshare.30122284 (ref. ^44^).

## Code availability

All custom R scripts used for statistical analyses and figure generation in this study have been deposited in the Figshare repository and are fully publicly accessible under accession code 10.6084/m9.figshare.30122284 (ref. ^44^).
