# Supplementary material for: A universal scaling law of intra-urban inequality
Source: Nat Commun. 2026 May 9;17:6264. doi: 10.1038/s41467-026-73015-1 (PMC13376171; doi:10.1038/s41467-026-73015-1)
Supplement: Supplementary file 1 — Supplementary Information [file 41467_2026_73015_MOESM1_ESM.pdf]

Supplementary Information

A universal scaling law of intra-urban inequality

Conghong Huang[1,2#], Xiaodan Liu[1#], Shibin Zhang[1], Zongyang Jin[1], Nan Xu[3,4,5*], Weixin Ou[1,2*]

[1]College of Land Management, Nanjing Agricultural University, Nanjing 210095, China; [2]National & Local Joint Engineering, Research Center for Rural Land Resources Use and Consolidation, Nanjing 210095, China; [3]Key Laboratory for Geo-Environmental Monitoring of Great Bay Area, Ministry of Natural Resources, Shenzhen University, Shenzhen, China; [4]Guangdong Key Laboratory of Urban Informatics, Shenzhen University, Shenzhen, China; [5]School of Architecture and Urban Planning, Shenzhen University, Shenzhen, China.

** Correspondence to:*

Nan Xu (xunan2025@szu.edu.cn),

Weixin Ou (owx@njau.edu.cn)

List of Supplementary Items

Supplementary Figure 1: Global distribution of urban samples and group counts .... Page 3

Supplementary Figure 2: Robustness test of the scaling law: Quantile regression analysis ... Page 4

Supplementary Figure 3: Sensitivity analysis of different Gini coefficient calculation radii .... Page 5

Supplementary Figure 4: The effect of urban form on the residuals of the inequality scaling law .... Page 6

Supplementary Figure 5: The association between urban form metrics and the residuals of inequality scaling laws .... Page 7

Supplementary Figure 6: Correlations between different types of inequality .... Page 8

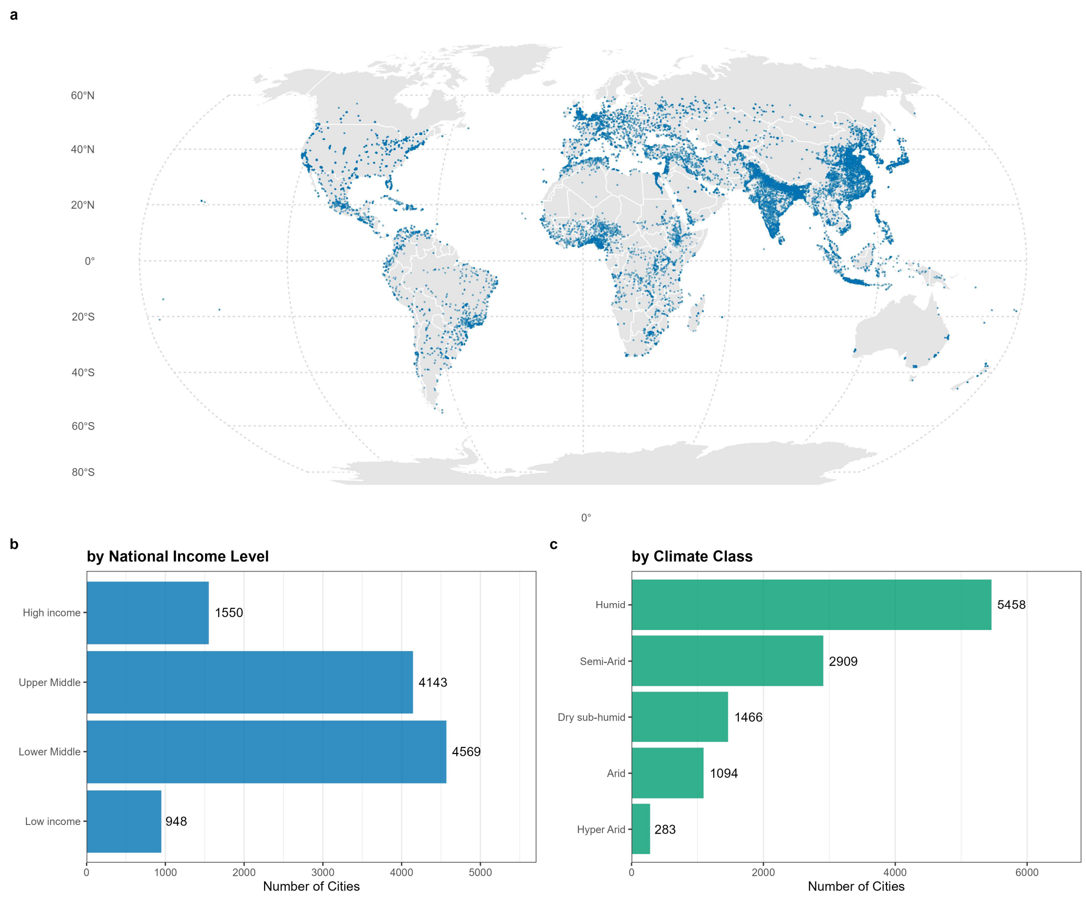

Supplementary Figure 1 Global distribution of urban samples and group counts. Overview of the study's data foundation. **a**, The global distribution of the cities (urban cores) included in the final analysis, plotted on a Robinson projection. **b**, Bar charts showing the number of cities within each national income level category. **c**, Bar charts showing the number of cities within each major climate class category.

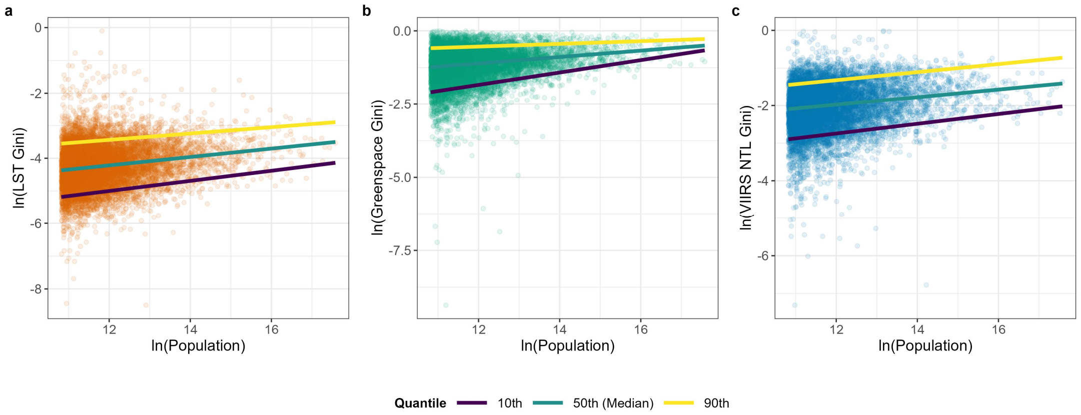

Supplementary Figure 2 Robustness test of the scaling law: Quantile regression analysis. Quantile regression analysis demonstrating the robustness of the scaling laws across different segments of the inequality distribution. The plots show the scaling relationships for the 10th (most equal cities), 50th (median), and 90th (most unequal cities) quantiles for (**a**) thermal inequality, (**b**) green space inequality, and (**c**) economic inequality. The general parallelism of the lines indicates that the scaling exponent is largely consistent across the distribution.

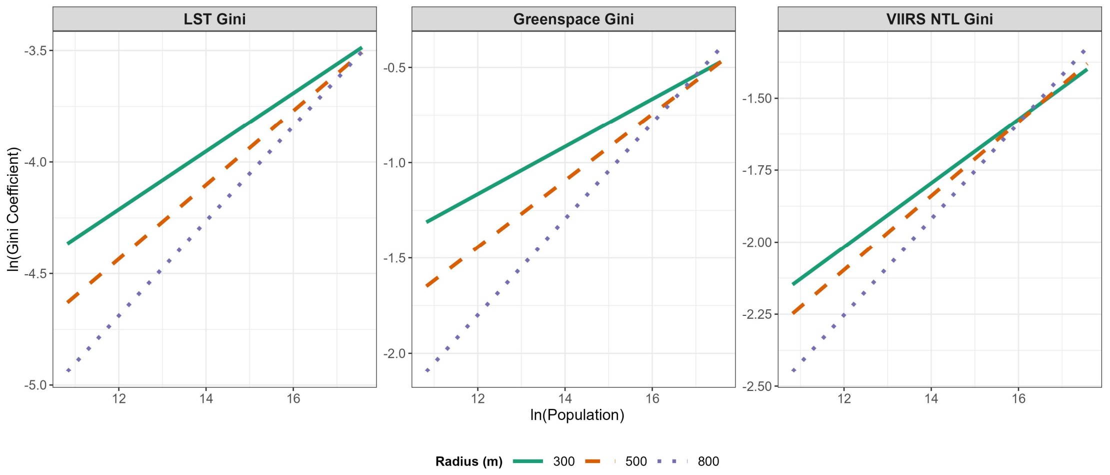

Supplementary Figure 3 Sensitivity analysis of different Gini coefficient calculation radii. The scaling laws of urban inequality are robust to the spatial radius used for Gini coefficient calculation. The plots show that while the scaling exponents (slopes) vary with the radius, all radii consistently demonstrate a super-linear scaling relationship ($\alpha > 0$). Notably, the lines for different radii converge as population size increases. This convergence suggests that while the absolute measure of inequality is sensitive to the calculation radius in smaller cities, this sensitivity diminishes in larger cities where inequality patterns are more established at a macro scale. This reinforces the finding that the exacerbation of inequality with urban growth is a fundamental property, independent of the specific neighborhood scale analyzed.

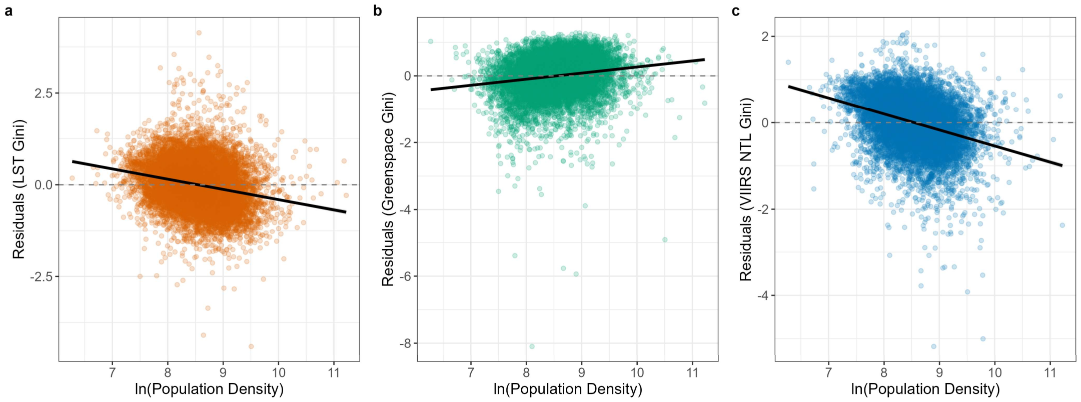

Supplementary Figure 4 The effect of urban form on the residuals of the inequality scaling law. Analysis of the residuals from the global scaling models (Figure 2) as a function of urban form, proxied by the natural logarithm of population density. A positive residual indicates a city is more unequal than predicted by its population size alone. The plots show the relationship for the residuals of (**a**) thermal inequality, (**b**) green space inequality, and (**c**) economic inequality. The solid line is the OLS regression fit.

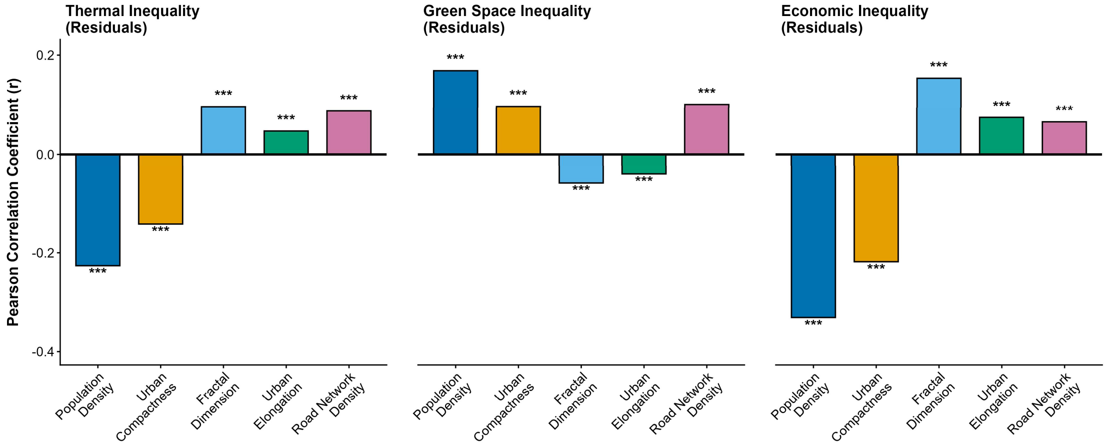

Supplementary Figure 5 The association between urban form metrics and the residuals of inequality scaling laws. The bar chart displays the Pearson correlation coefficients (*r*) between five key urban form indicators (Population Density, Urban Compactness, Fractal Dimension, Urban Elongation, and Road Network Density) and the residuals of the global scaling models for thermal, green space, and economic inequality. The residuals represent the deviation of a city's inequality from the value predicted by its population size alone. Asterisks denote statistical significance levels (*** $P < 0.001$, ** $P < 0.01$, * $P < 0.05$). The results indicate that compact and dense urban forms are generally associated with lower thermal and economic inequality (negative correlations) but higher green space inequality (positive correlations), highlighting a potential trade-off in urban planning.

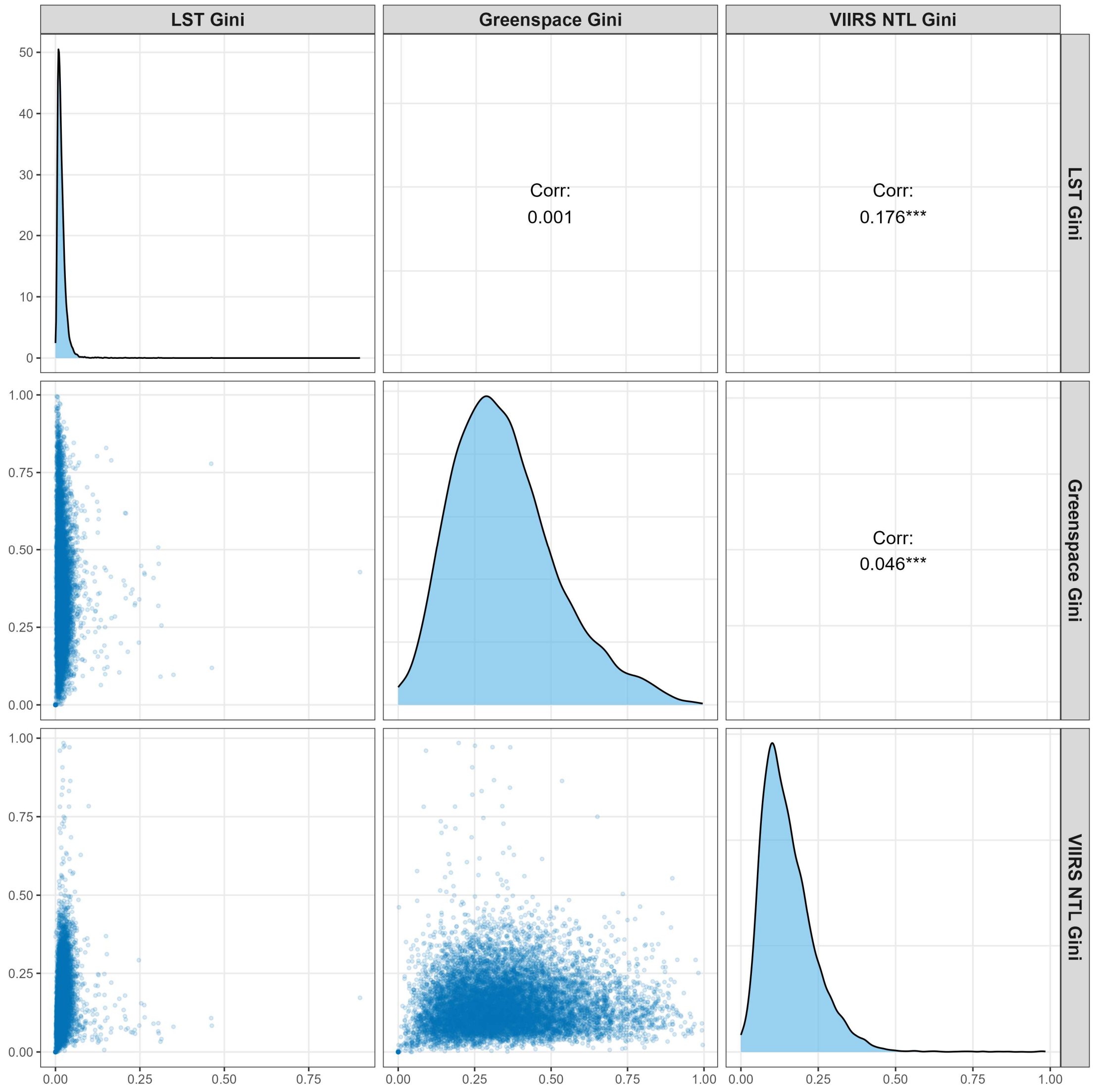

Supplementary Figure 6 Correlations between different types of inequality. A scatterplot matrix showing the pairwise relationships between the three Gini coefficients. The diagonal panels show the probability density distribution of each variable. The lower-triangular panels show the scatter plots. The upper-triangular panels show the Pearson correlation coefficient (r) with its statistical significance.
