# Supplementary material for: A universal scaling law of intra-urban inequality
Source: Nat Commun. 2026 May 9;17:6264. doi: 10.1038/s41467-026-73015-1 (PMC13376171; doi:10.1038/s41467-026-73015-1)
Supplement: Supplementary file 2 — Reporting Summary [file 41467_2026_73015_MOESM2_ESM.pdf]

nature portfolio

Corresponding author(s): Weixin Ou

Last updated by author(s): Mar 30, 2025

Reporting Summary

Nature Portfolio wishes to improve the reproducibility of the work that we publish. This form provides structure for consistency and transparency in reporting. For further information on Nature Portfolio policies, see our Editorial Policies and the Editorial Policy Checklist.

Statistics

For all statistical analyses, confirm that the following items are present in the figure legend, table legend, main text, or Methods section.

n/a	Confirmed	
☐	☒	The exact sample size (*n*) for each experimental group/condition, given as a discrete number and unit of measurement
☐	☒	A statement on whether measurements were taken from distinct samples or whether the same sample was measured repeatedly
☐	☒	The statistical test(s) used AND whether they are one- or two-sided<br>*Only common tests should be described solely by name; describe more complex techniques in the Methods section.*
☐	☒	A description of all covariates tested
☐	☒	A description of any assumptions or corrections, such as tests of normality and adjustment for multiple comparisons
☐	☒	A full description of the statistical parameters including central tendency (e.g. means) or other basic estimates (e.g. regression coefficient) AND variation (e.g. standard deviation) or associated estimates of uncertainty (e.g. confidence intervals)
☐	☒	For null hypothesis testing, the test statistic (e.g. *F*, *t*, *r*) with confidence intervals, effect sizes, degrees of freedom and *P* value noted<br>*Give P values as exact values whenever suitable.*
☒	☐	For Bayesian analysis, information on the choice of priors and Markov chain Monte Carlo settings
☒	☐	For hierarchical and complex designs, identification of the appropriate level for tests and full reporting of outcomes
☐	☒	Estimates of effect sizes (e.g. Cohen's *d*, Pearson's *r*), indicating how they were calculated

Our web collection on statistics for biologists contains articles on many of the points above.

Software and code

Policy information about availability of computer code

Data collection	All primary remote sensing data were acquired and processed on the Google Earth Engine (GEE) platform. This included the calculation of NDVI from Sentinel-2 imagery and Land Surface Temperature (LST) from Landsat 8/9 data. The LST calculation was implemented in GEE following a previously published open-source algorithm (Ref 39). We also used QGIS (version 3.34) for the visual validation of the NDVI threshold.
Data analysis	All statistical analyses were conducted in the R programming environment (version 4.5.1). Specifically, the population-weighted Gini coefficients were computed in R using the gini.wtd function from the dineq package. All statistical tests, including the Ordinary Least Squares (OLS) regressions, interaction analyses, and quantile regressions, were performed using custom R scripts. These scripts are publicly available at the Figshare repository (https://doi.org/10.6084/m9.figshare.30122284), as cited in our manuscript.

For manuscripts utilizing custom algorithms or software that are central to the research but not yet described in published literature, software must be made available to editors and reviewers. We strongly encourage code deposition in a community repository (e.g. GitHub). See the Nature Portfolio guidelines for submitting code & software for further information.

Data

Policy information about availability of data

All manuscripts must include a data availability statement. This statement should provide the following information, where applicable:

- Accession codes, unique identifiers, or web links for publicly available datasets
- A description of any restrictions on data availability
- For clinical datasets or third party data, please ensure that the statement adheres to our policy

The third-party datasets used in this study are publicly available. The Global Human Settlement Urban Centre Database (GHS-UCDB) is available from the European Commission's Joint Research Centre. Satellite imagery from Sentinel-2, thermal data from Landsat 8/9, and the VIIRS Nighttime Lights data were sourced from the Google Earth Engine data catalog. These datasets are provided by the European Space Agency, the U.S. Geological Survey, and the Earth Observation Group at the Payne Institute for Public Policy, respectively. The WorldPop population data are available at worldpop.org. The processed, city-level dataset generated in this study, which includes the source data underlying all main and supplementary figures (provided as dataMerged.csv and dataMerged.rds), has been deposited in the Figshare database under accession code https://doi.org/10.6084/m9.figshare.30122284.

Research involving human participants, their data, or biological material

Policy information about studies with human participants or human data. See also policy information about sex, gender (identity/presentation), and sexual orientation and race, ethnicity and racism.

Reporting on sex and gender	Not applicable. This study analyzes aggregate population counts and remote sensing data. It does not use or have access to individual-level demographic data such as sex or gender.
Reporting on race, ethnicity, or other socially relevant groupings	The socially relevant categorization variable used in our analysis is National Income Level. We used the four-category classification (High, Upper Middle, Lower Middle, and Low Income) provided directly by the Global Human Settlement Urban Centre Database (GHS-UCDB). This administrative grouping was used to stratify our global sample to test for socioeconomic modulation of the scaling laws.
Population characteristics	Not applicable. This study does not involve human research participants. The study's "population" consists of 11,210 urban centers, not individuals.
Recruitment	Not applicable. No human participants were recruited for this study.
Ethics oversight	Not applicable. This study was based entirely on publicly available, anonymous, and aggregated datasets (e.g., satellite imagery and public census/population databases). It did not involve human subjects and therefore did not require ethics committee approval.

Note that full information on the approval of the study protocol must also be provided in the manuscript.

Field-specific reporting

Please select the one below that is the best fit for your research. If you are not sure, read the appropriate sections before making your selection.

☐ Life sciences ☐ Behavioural & social sciences ☒ Ecological, evolutionary & environmental sciences

For a reference copy of the document with all sections, see nature.com/documents/nr-reporting-summary-flat.pdf

Ecological, evolutionary & environmental sciences study design

All studies must disclose on these points even when the disclosure is negative.

Study description	This is a global, cross-sectional, quantitative study of 11,210 urban centers. It uses Ordinary Least Squares (OLS) regression on log-transformed data to model the scaling relationship between city population (independent variable) and three types of intra-urban inequality (dependent variables: LST Gini, Green Space Gini, NTL Gini) . The study design is stratified, and it explicitly tests for interaction effects by classifying cities based on two main factors: national income level (4 levels) and background climate class (5 levels).
Research sample	The research sample consists of 11,210 urban centers worldwide. The unit of analysis is the city, not a biological organism. The primary data source for defining these urban centers is the Global Human Settlement Urban Centre Database (GHS-UCDB, R2024A). This sample is intended to represent a globally consistent and comprehensive set of urban areas. Data for inequality metrics were derived from multiple publicly available satellite data sources, including Sentinel-2 (green space) , Landsat 8/9 (LST) , and VIIRS (nighttime lights).
Sampling strategy	No sample-size calculation was performed, as this study used a comprehensive, global-scale dataset rather than a small sample. We aimed to include all urban centers available in the Global Human Settlement Urban Centre Database (GHS-UCDB, R2024A), which initially contained 11,422 locations. The final sample size of 11,210 cities was determined by data availability, excluding entries with missing or non-positive values. This large N is sufficient for robustly establishing global scaling laws and testing statistical interactions.
Data collection	Data was not collected in the field by the authors. The research team acquired and processed all data from publicly available, archival

remote sensing and geospatial datasets. Data processing was conducted by the authors on the Google Earth Engine (GEE) platform. This involved calculating composite imagery for green space (Sentinel-2) , LST (Landsat 8/9) , and economic activity (VIIRS) for the period 2022-2024. Population-weighted Gini coefficients were then calculated by the authors in R.

Timing and spatial scale	The study is global in spatial scale, covering 11,210 urban centers. The remote sensing data are composites from a recent period: Green space (NDVI) was from the 2024 growing season; Land Surface Temperature (LST) and Nighttime Lights (NTL) were averaged from 2022–2024. The analysis of inequality was performed at a 100m grid resolution, with neighborhood-level exposure calculated using a 500m radius buffer.
Data exclusions	Yes, data were excluded. The analysis began with the 11,422 urban centers in the GHS-UCDB R2024A. We excluded 212 centers (11,422 - 11,210) from the final analysis. The pre-established rationale for this exclusion was to remove any urban centers that had missing or non-positive values for any of the key variables (population, LST, green space, or nighttime lights).
Reproducibility	Reproducibility was ensured by making the processed, city-level dataset (serving as the source data) and all R scripts used for analysis publicly available in a Figshare repository (https://doi.org/10.6084/m9.figshare.30122284).
Randomization	Not applicable. This was not a randomized controlled experiment. The allocation of urban centers into analytical groups (e.g., 'Low Income' or 'Arid') was not performed by the researchers. Instead, cities were stratified based on their pre-existing, non-random attributes (national income level and background climate class).
Blinding	Not applicable. Blinding was not relevant to this study. The analysis involved computational processing of objective, publicly available geospatial and remote sensing data. There was no subjective assessment or experimental treatment involved that would require blinding.

Did the study involve field work? ☐ Yes ☒ No

Reporting for specific materials, systems and methods

We require information from authors about some types of materials, experimental systems and methods used in many studies. Here, indicate whether each material, system or method listed is relevant to your study. If you are not sure if a list item applies to your research, read the appropriate section before selecting a response.

Materials & experimental systems

n/a	Involved in the study
☒	☐ Antibodies
☒	☐ Eukaryotic cell lines
☒	☐ Palaeontology and archaeology
☒	☐ Animals and other organisms
☒	☐ Clinical data
☒	☐ Dual use research of concern
☒	☐ Plants

Methods

n/a	Involved in the study
☒	☐ ChIP-seq
☒	☐ Flow cytometry
☒	☐ MRI-based neuroimaging

Plants

Seed stocks	*Report on the source of all seed stocks or other plant material used. If applicable, state the seed stock centre and catalogue number. If plant specimens were collected from the field, describe the collection location, date and sampling procedures.*
Novel plant genotypes	*Describe the methods by which all novel plant genotypes were produced. This includes those generated by transgenic approaches, gene editing, chemical/radiation-based mutagenesis and hybridization. For transgenic lines, describe the transformation method, the number of independent lines analyzed and the generation upon which experiments were performed. For gene-edited lines, describe the editor used, the endogenous sequence targeted for editing, the targeting guide RNA sequence (if applicable) and how the editor was applied.*
Authentication	*Describe any authentication procedures for each seed stock used or novel genotype generated. Describe any experiments used to assess the effect of a mutation and, where applicable, how potential secondary effects (e.g. second site T-DNA insertions, mosiacism, off-target gene editing) were examined.*
