# Supplementary material for: A universal scaling law of intra-urban inequality
Source: Nat Commun. 2026 May 9;17:6264. doi: 10.1038/s41467-026-73015-1 (PMC13376171; doi:10.1038/s41467-026-73015-1)
Supplement: Supplementary file 3 — Transparent Peer Review file [file 41467_2026_73015_MOESM3_ESM.pdf]

nature portfolio

Peer Review File

A universal scaling law of intra-urban inequality

Corresponding Author: Professor Weixin Ou

This file contains all reviewer reports in order by version, followed by all author rebuttals in order by version.

Version 0:

Reviewer comments:

Reviewer #1

(Remarks to the Author)
This article explores intra-urban inequalities in terms of heat island, green spaces and economic activity for 11,000 urban centres around the world, based on data from multiple sources. The authors are to be commended for introducing environmental variables that have rarely been considered in the examination of scale effects in cities. The results show that inequalities are systematically greater in large cities, with an increase of just under ten per cent when considering cities that are twice as large.
However, from the abstract onwards, the authors show a tendency to interpret their cross-sectional observations in a longitudinal manner (‘as cities grow, they become systematically more unequal’; ‘urban growth inherently fosters inequality’). This error has already been challenged in the literature (Pumain D. 2012, Urban systems dynamics, urban growth and scaling laws: the question of ergodicity, in J. Portugali and H. Meyer, with E. Stolk and E. Tan, Eds. Complexity Theories of Cities Have Come of Age: An overview with implications to urban planning and design. Springer, Heidelberg, Berlin, 93-106). However, given that current inequalities are less pronounced in rich countries than in less developed countries, it is conceivable that future urban growth will correct the trend towards growing inequalities as countries achieve a higher standard of living. Hopefully.
While acknowledging the considerable work accomplished by the authors in gathering and processing a vast set of data, one might ask them to tone down those claims that shift from cross-sectional to longitudinal interpretations, which could be considered misinterpretations, and to add a paragraph specifying some of the limitations of their work before it can be used in urban planning, for example due to the use of satellite images of night-time lights as a measure of economic activity.

Reviewer #2

(Remarks to the Author)
This is a very relevant paper that potentially unveils some very interesting and relevant theoretical and practical considerations on urban inequality. It is very well written overall, And it was insightful to read on some critical findings such as “The scaling exponents for these inequalities are substantially steeper in low-income countries compared to high- and upper-middle-income nations. This suggests that as cities in developing nations grow, they face a much more rapid intensification of spatial disparities in both ecological amenities and economic activity. This aligns with theories of uneven development and provides large-scale empirical evidence that the capacity to manage and distribute resources equitably scales with national wealth.” However, I think that the paper dilutes (and at times fully overlooks) some other relevant considerations that merit reflection. Details on these are provided below.

Main
Explain ‘power-law scaling relationships’ more clearly. What does it mean to ‘scale linearly’ or to ‘scale super-linearly’? Or what does it mean ‘suggesting increasing returns to scale, while infrastructure scales sub-linearly, indicating economies of scale1 The non-technical reader needs to be able to understand these terms.
The introduction fails to better contextualise the importance of thermal comfort, green space access and night light (as a proxy to economic development) with regards to human health and wellbeing. Also, the paper fails to justify why these three parameters have been selected and not others.

Results
How were the ‘urban cores’ defined? What is the underlaying dataset? Does the urban areas encompass both intensive and dispersed urban fabric? This needs to be explained for methodological transparency. I have read the details in the methods

section then, but a cross-reference to that section or a simple definition of urban core at this point would facilitate a clearer and nuanced understanding of the results.
Similarly, the national income level and the climate class breaks in Figure S1 are not explained nor justified which needs addressing. This also applies to Figure 5. Again, I can read about these in the methods section (see related comments there) but a cross-reference is needed for clarity.
“While population size serves as a fundamental predictor, we anticipate that its explanatory power for the variance in inequality will be modest, pointing to the critical role of other modulating factors” – Why is this anticipated and population size ruled out as a determinant of inequality? This sentence needs to be both reconsidered and justified.
One important factor to consider, is the spatial footprint of urban centres when it comes to inequalities. The more dense the cities, the less green spaces for example, and thus the more potential for inequalities in access to these. Similarly, the more dense the cities, the more likely the heat urban effect due to street canyon structures and less green spaces and trees. These considerations are overlooked in the introduction to the paper and should be given consideration to strengthen the narrative and acknowledge the caveats In the findings. They are considered in the discussion when ‘population density’ is referred to, but a more coherent account of the geographical dimension of inequalities is needed.
“This finding suggests that the physical constraints of the environment, likely related to water availability for evaporative cooling, play a key role in shaping thermal disparities as cities grow” – This is a relevant findings indeed, but as noted above, there is also the likelihood that urban form (which is rather different in different climatic conditions and influences the urban structure overall) significantly impact on heat island effects. Something similar then applies to “indicating a more rapid exacerbation of green space inequity in wetter regions once a city reaches a certain size” in relation to the comments above regarding urban structure and the density of cities consideration highlighted above. I think that such ‘confounding’ factors must be acknowledged and be part of the discussion.
“Our findings are robust to methodological choices.” Which remain unexplained and unjustified on page 10. A cross-reference to the methods section here would be helpful.
“urban form, proxied by population density, explains a significant portion of the remaining variance in inequality” – I am unconvinced that urban form can be so bluntly proxied by population density. While this holds true for definite urban cores/areas, this generalisation underestimates the inequality inherent in urban form and population density themselves with richer areas typically having lower density and more open urban forms. I understand that the paper is trying to look at the issue from a global and strategic perspective, but these other considerations merit their acknowledgement and reflection.
Figure 1 – it is not immediately clear what the conceptual schematics mean to illustrate. The right/left split is misleading as inequalities are never so defined in urban areas; these can take the form of pockets rather than such radical splits. Also, the green/yellow/orange backgrounds are not explained and add to the confusion.

Discussion
“predictive science capable of forecasting the evolution of inequality as a function of urban growth.” Yet the degree of inequality is very context specific, and influenced and modulated by many factors which again, the paper fails to acknowledge.
The discussion is otherwise well articulated and some relevant and important points are made (e.g. “For instance, the finding that low-income nations are on a steeper trajectory of green space inequality highlights the urgent need for well-funded policies that prioritize equitable greening strategies (…)”.
“our analysis is cross-sectional and does not capture the temporal evolution of inequality within individual cities” – the year of the data this study was based on is not acknowledged. This only comes in the methods section but acknowledging this from the outset (e.g. results section) would strengthen the validity of the results and discussion by providing a clear and current timeframe to the reader.
The limitations paragraph of the discussion section should address some of the aspects raised above (e.g. urban footprint, urban form, etc.).

Methods
The methods section needs to justify why these three parameters are examined in the first place (see related comment above).
“The socioeconomic stratification was based on the four national income levels (High, Upper Middle, Lower Middle, and Low Income) provided by the GHS-UCDB.” Provide a reference to back this up and include an outline of income breaks behind those categories for clarity.

Version 1:

Reviewer comments:

Reviewer #1

(Remarks to the Author)
The authors took my suggestions on the first version of their article very well into account. They clarified the vocabulary without confusing cross-sectional and longitudinal analyses. The limitations of the study due to the nature of the data were also clearly stated. I believe this second version is ready for publication.

Reviewer #2

(Remarks to the Author)
I am satisfied that the authors have appropriately addressed my comments by clarifying certain considerations and acknowledging important research shortcomings and confounding factors. The article reads better now and the description

of the methodology and results is more comprehensive.

Open Access This Peer Review File is licensed under a Creative Commons Attribution 4.0 International License, which permits use, sharing, adaptation, distribution and reproduction in any medium or format, as long as you give appropriate credit to the original author(s) and the source, provide a link to the Creative Commons license, and indicate if changes were made.
In cases where reviewers are anonymous, credit should be given to 'Anonymous Referee' and the source.
The images or other third party material in this Peer Review File are included in the article's Creative Commons license, unless indicated otherwise in a credit line to the material. If material is not included in the article's Creative Commons license and your intended use is not permitted by statutory regulation or exceeds the permitted use, you will need to obtain permission directly from the copyright holder.
To view a copy of this license, visit https://creativecommons.org/licenses/by/4.0/

Responses to the reviewers

Reviewer #1 (Remarks to the Author):

Comment 1: This article explores intra-urban inequalities in terms of heat island, green spaces and economic activity for 11,000 urban centres around the world, based on data from multiple sources. The authors are to be commended for introducing environmental variables that have rarely been considered in the examination of scale effects in cities. The results show that inequalities are systematically greater in large cities, with an increase of just under ten per cent when considering cities that are twice as large.

Response: We sincerely thank the Reviewer for the encouraging comments and for recognizing the value of introducing environmental variables into the examination of urban scale effects. We appreciate the acknowledgment of the extensive data processing work involved in analyzing over 11,000 urban centers. The reviewer's positive assessment of our key finding, that inequalities increase by approximately 10% when comparing cities that are twice as large, further motivates our investigation into the underlying mechanisms of this scaling relationship.

Comment 2: However, from the abstract onwards, the authors show a tendency to interpret their cross-sectional observations in a longitudinal manner (‘as cities grow, they become systematically more unequal’; ‘urban growth inherently fosters inequality’). This error has already been challenged in the literature (Pumain D. 2012, Urban systems dynamics, urban growth and scaling laws: the question of ergodicity, in J. Portugali and H. Meyer, with E. Stolk and E. Tan, Eds. Complexity Theories of Cities Have Come of Age: An overview with implications to urban planning and design. Springer, Heidelberg, Berlin, 93-106). However, given that current inequalities are less pronounced in rich countries than in less developed countries, it is conceivable that future urban growth will correct the trend towards growing inequalities as countries achieve a higher standard of living. Hopefully.

Response: Thank you very much for this valuable suggestion. We fully accept the Reviewer’s critical methodological insight regarding the distinction between cross-sectional observations and longitudinal evolution. We fully agree that cross-sectional data cannot simply substitute for the temporal dynamics of individual cities, and we appreciate the reference to the ergodicity problem discussed in Pumain (2012). Indeed, cross-sectional patterns across cities of different sizes at a single point in time cannot be directly interpreted as the temporal trajectory of any individual city.

To address this issue, we have systematically revised the manuscript to remove any language that could imply a temporal interpretation of our cross-sectional findings. Specifically:

In the Abstract and Introduction section, we have replaced dynamic phrasing such as *"as cities grow"* and *"urban growth inherently fosters inequality"* with static, cross-sectional descriptions: *"larger cities exhibit systematically higher inequality"* and *"inequality scales with city size."*

In the Discussion section, we have added a dedicated Limitations paragraph that explicitly frames our findings as characterizing the *"macro-structural state of the global urban system"* rather than predicting future trajectories. This section now cites Pumain (2012) alongside Depersin and Barthelemy (2018) and Keuschnigg et al. (2019) to acknowledge that longitudinal scaling exponents may diverge from cross-sectional ones due to path dependencies.

The specific revisions are detailed as follows.

"Cities are engines of the global economy and innovation, yet their rapid development is often accompanied by rising social inequality. While

many aggregate urban attributes, such as economic output and infrastructure, follow predictable scaling laws with population size, a unifying theoretical framework and systematic empirical evidence for how intra-urban inequality ***scales with city size*** *have been lacking. To bridge this critical knowledge gap, we constructed a comprehensive dataset for over 11,000 urban centers worldwide and used multi-source satellite remote sensing datasets to quantify intra-urban inequality in three dimensions: thermal exposure, green space distribution, and economic activity. Here we show that all three forms of inequality are not random but follow a universal super-linear scaling law with city population, indicating that* ***inequality is systematically higher in larger cities****. Specifically, a doubling of city population is associated with an approximate 9% (scaling exponent* $\alpha = 0.13$*), 9% (*$\alpha = 0.12$*), and 8% (*$\alpha = 0.11$*) increase in the Gini coefficients for thermal, green space, and economic inequality, respectively. Further analysis reveals that these scaling laws are significantly modulated by national socioeconomic development and background climate conditions. Most notably, the* ***scaling*** *of green space and economic inequality is far more rapid in low-income nations, where the scaling exponents are more than double those of high-income countries. Meanwhile, the scaling of thermal inequality is most acute in arid and semi-arid climates. These findings provide the first large-scale, quantitative evidence that the distribution of urban inequality*

is a predictable and systematic property intrinsically linked to city size. This research elevates the study of urban environmental justice from case-specific descriptions to a predictive, theory-based science. Our findings suggest that without targeted interventions, ***urban scale is structurally associated with higher*** *inequality, challenging the visions of urban equity outlined in the UN's Sustainable Development Goals. This work provides a quantitative framework to* ***characterize*** *inequality* ***scaling*** *and inform proactive urban planning."* ***(Page 2-3, Line 21-46)***

In Discussion section

"Our study has several limitations. First, our scaling analysis is based on cross-sectional data. While this approach effectively characterizes the macro-structural state of the global urban system[17]*, it should not be conflated with the longitudinal trajectories of individual cities.* ***As noted by Pumain***[33]***, Depersin and Barthelemy***[18]***, and Keuschnigg et al.***[19]***, longitudinal scaling exponents may diverge from cross-sectional ones due to path dependencies****. Second, regarding urban form, we used population density as a primary proxy. Although we validated this against geometric metrics (e.g., compactness, fractal dimension) and found density to be the most robust predictor of inequality residuals (Supplementary Fig. S5), we acknowledge that density alone does not capture the full complexity*

of urban morphology (e.g., street canyon effects on heat trapping). Third, we use remote sensing-based proxies for complex phenomena; LST is not equivalent to human thermal comfort, and Nighttime Lights capture only a facet of economic activity. Fourth, our Gini coefficient approach quantifies overall inequality but does not reveal the specific social groups (e.g., defined by race or ethnicity) who are most affected. Finally, our scaling analysis reveals strong correlations, but establishing the precise causal mechanisms requires further, more detailed investigation, potentially integrating micro-level data as has been demonstrated by Arvidsson et al.[20]*"*

(Page 15-16, Line 304-320)

In addition, we appreciate the Reviewer's observation that inequalities are less pronounced in rich countries, and the hopeful suggestion that economic development may ultimately temper the relationship between city size and inequality. This point aligns well with our findings: as we now emphasize in the Discussion, the flatter scaling exponents observed for high-income nations (Figure 3) support the view that economic development can indeed mitigate inequality.

We believe these changes appropriately contextualize our findings while preserving the substantive contribution of identifying a robust cross-sectional scaling law.

Comment 3: While acknowledging the considerable work accomplished by the authors in gathering and processing a vast set of data, one might ask them to tone down those claims that shift from cross-sectional to longitudinal interpretations, which could be considered misinterpretations, and to add a paragraph specifying some of the limitations of their work before it can be used in urban planning, for example due to the use of satellite images of night-time lights as a measure of economic activity.

Response: Thank you very much for this valuable suggestion. We have followed the Reviewer's suggestion to tone down our claims and explicitly address the study's constraints. Following their advice, we have taken two specific actions to address the concern about potential overinterpretation. Specifically, we have added a comprehensive Limitations section at the end of the Discussion to caution against overinterpretation in urban planning contexts. The revised Limitations section provides a detailed discussion of the constraints associated with our remote sensing proxies:

"Our study has several limitations. First, our scaling analysis is based on cross-sectional data. While this approach effectively characterizes the macro-structural state of the global urban system[17], it should not be conflated with the longitudinal trajectories of individual cities. As noted by

Pumain[33], Depersin and Barthelemy[18], and Keuschnigg et al.[19], longitudinal scaling exponents may diverge from cross-sectional ones due to path dependencies. Second, regarding urban form, we used population density as a primary proxy. Although we validated this against geometric metrics (e.g., compactness, fractal dimension) and found density to be the most robust predictor of inequality residuals (Supplementary Fig. S5), we acknowledge that density alone does not capture the full complexity of urban morphology (e.g., street canyon effects on heat trapping). Third, we use remote sensing-based proxies for complex phenomena; LST is not equivalent to human thermal comfort, and Nighttime Lights capture only a facet of economic activity. Fourth, our Gini coefficient approach quantifies overall inequality but does not reveal the specific social groups (e.g., defined by race or ethnicity) who are most affected. Finally, our scaling analysis reveals strong correlations, but establishing the precise causal mechanisms requires further, more detailed investigation, potentially integrating micro-level data as has been demonstrated by Arvidsson et al.[20]"

(Page 15-16, Line 304-320)

We sincerely thank you again for all your comments, which have significantly improved the quality of this manuscript. In the revised manuscript, all changes are highlighted in yellow. Please refer to the revised details within the text. If any issues remain to be addressed, we

would appreciate the opportunity to further revise and improve the manuscript.

Reviewer #2 (Remarks to the Author):

Comment 1 (General): This is a very relevant paper that potentially unveils some very interesting and relevant theoretical and practical considerations on urban inequality. It is very well written overall, And it was insightful to read on some critical findings such as “The scaling exponents for these inequalities are substantially steeper in low-income countries compared to high- and upper-middle-income nations. This suggests that as cities in developing nations grow, they face a much more rapid intensification of spatial disparities in both ecological amenities and economic activity. This aligns with theories of uneven development and provides large-scale empirical evidence that the capacity to manage and distribute resources equitably scales with national wealth.” However, I think that the paper dilutes (and at times fully overlooks) some other relevant considerations that merit reflection. Details on these are provided below.

Response: We are very grateful to the Reviewer for both the encouraging words and the constructive critique. It is reassuring to know that our core findings, especially the cross-national differences in scaling exponents and their alignment with uneven development theory, came across as insightful and well-supported.

At the same time, we fully accept the Reviewer's point that the original manuscript required more detailed attention to certain relevant considerations. This feedback has helped us identify areas where the manuscript could be strengthened. Below, we respond to each specific comment in turn, with particular attention to improving the justification for our variable choices and deepening the analysis of urban form.

Comment 2 (Main): Explain ‘power-law scaling relationships’ more clearly. What does it mean to ‘scale linearly’ or to ‘scale super-linearly’? Or what does it mean ‘suggesting increasing returns to scale, while infrastructure scales sub-linearly, indicating economies of scale1 The non-technical reader needs to be able to understand these terms.

Response: We thank the Reviewer for this helpful suggestion. We agree that scaling concepts should be accessible to readers from diverse backgrounds, including those outside the field of complex systems. We acknowledge that the original manuscript did not provide sufficient explanation of scaling concepts for non-specialist readers, and we agree that these terms should be accessible to a broader audience. To address this, we have revised the Introduction to provide a plain-language explanation of power-law scaling relationships. The revised contents are detailed as follows.

“Typically, socioeconomic outputs like wealth and innovation scale super-linearly (exponent > 1), ***meaning they grow faster than the population (suggesting increasing returns)****, while infrastructure scales sub-linearly, indicating economies of scale (****i.e., growing slower than population, resulting in material savings)****[13]. ”* ***(Page 5, Line 80-84)***

Comment 3: The introduction fails to better contextualise the importance of thermal comfort, green space access and night light (as a proxy to economic development) with regards to human health and wellbeing. Also, the paper fails to justify why these three parameters have been selected and not others.

Response: Thank you very much for this valuable suggestion. We agree that the Introduction, in its initial version, did not adequately justify our choice of variables nor sufficiently link them to human health and wellbeing. This was a significant omission, and we have addressed it thoroughly in the revised manuscript.

Specifically, we have substantially rewritten the second paragraph of the Introduction to:

1) Provide a clear theoretical rationale for selecting land surface temperature (LST), urban green space (UGS), and nighttime lights (NTL) as our three core dimensions of urban inequality;

2) Explicitly link each variable to established public health outcomes, drawing on recent epidemiological and urban health literature;

3) Demonstrate that these three dimensions are not arbitrary choices, but rather represent complementary and critical determinants of urban wellbeing.

The revised contents are detailed as follows.

"However, this progress is often accompanied by profound social and environmental costs. ***A critical but often overlooked factor is the spatial footprint of urbanization: while higher urban density drives economic agglomeration, it may inadvertently intensify local environmental stressors. For instance, densification presents significant challenges for preserving green spaces and maintaining microclimatic comfort[5], creating a "local health paradox" where the benefits of urban growth are unevenly distributed[6].***

To understand these trade-offs, we focus on three critical dimensions of the urban environment that are directly linked to human health and wellbeing: thermal exposure, green infrastructure, and economic activity. First, Land Surface Temperature (LST) serves as a key indicator of heat exposure risk. The uneven distribution of urban heat often creates a "landscape of thermal inequity," where marginalized populations are disproportionately exposed to heat stress[7–9]. Second, Urban Green Space (UGS) acts as a vital health resource. Systematic reviews confirm that access to green space significantly reduces all-cause mortality by mitigating heat islands, encouraging physical activity, and facilitating psychological restoration[10,11]. Third, Nighttime Light (NTL) intensity provides a robust high-resolution proxy for the intensity of economic activity at the micro-scale[12,13], reflecting the spatial distribution of economic wellbeing. *Consequently, understanding the fundamental principles that govern urban organization and its socio-ecological consequences is a critical scientific challenge for achieving sustainable urban development[1,14].”* ***(Page 4-5, Line 54-75)***

Comment 4 (Results): How were the ‘urban cores’ defined? What is the underlaying dataset? Does the urban areas encompass both intensive and dispersed urban fabric? This needs to be explained for methodological transparency. I have read the details in the methods section then, but a

cross-reference to that section or a simple definition of urban core at this point would facilitate a clearer and nuanced understanding of the results.

Response: Thank you very much for this valuable suggestion. We agree that methodological transparency requires a clear definition of "urban cores," and we have revised the manuscript accordingly. In the Methods, we now specify that urban centers are defined using the GHS-UCDB criteria: contiguous grid cells with a density of at least 1,500 inhabitants/km^2 and a total population of at least 50,000. This definition captures high-density urban cores and excludes dispersed low-density areas, ensuring that our analysis focuses on the intensive urban fabric where inequality dynamics are most relevant. Moreover, to help readers interpret the results, we have also added a cross-reference in the Results section, directing them to the Methods for full details.

The specific revisions are detailed as follows.

In Methods section

*"Our study is based on a final sample of 11,210 urban centers worldwide, with broad coverage across different socioeconomic and climatic regions (**Supplementary Fig. S1**). To ensure a globally consistent and comparable definition of urban areas, we utilized the Global Human Settlement Urban*

Centre Database version R2024A (GHS-UCDB)[34,35], which initially contained data for 11,422 locations. ***In this database, an Urban Centre is defined as a cluster of contiguous grid cells with a density of at least 1,500 inhabitants per km² and a total population of at least 50,000, representing the high-density urban core.”*** ***(Page 17, Line 333-340)***

In Results section

*“Our analysis, encompassing over 11,000 cities worldwide (**based on data circa 2024, defined as high-density urban centers, see Methods**).”* ***(Page 7, Line 132-133)***

We believe these additions provide the clarity and transparency the Reviewer requested.

Comment 5 (Results): Similarly, the national income level and the climate class breaks in Figure S1 are not explained nor justified which needs addressing. This also applies to Figure 5. Again, I can read about these in the methods section (see related comments there) but a cross-reference is needed for clarity.

Response: Thank you very much for this valuable suggestion. To improve clarity and transparency, we have added cross-references in the Results

section pointing to the Methods for details on both income and climate classifications. In the Methods, we now explicitly justify the classification breaks by citing the World Bank income thresholds (fiscal year 2024) and the UNEP Aridity Index categories. The revised Methods text includes the specific GNI per capita ranges and AI thresholds used. These additions ensure that readers can easily locate and understand the basis for the stratifications in Figures S1 and 5. The revised contents are detailed as follows.

*"The analysis stratified by national income level (**see Methods for income classification details**) reveals significant divergence in the scaling of both green space and economic inequality (Fig. 3)." (**Page 9, Line 161-163**)*

*"Background climate also modulates these scaling laws (**see Methods for climate classification details**),…" (**Page 9, Line 177-178**)*

*"The socioeconomic stratification was based on the four national income levels provided by the GHS-UCDB, **which aligns with the World Bank classification (fiscal year 2024) based on 2022 GNI per capita: Low Income (<= $1,135), Lower-Middle Income ($1,136–$4,465), Upper-***

Middle Income ($4,466–$13,845), and High Income (> $13,845)*[41]*.”

(Page 21, Line 429-433)

“The climatic stratification was based on the Aridity Index (AI) from the Global-AI_ET0_v3 dataset42, which was classified into five major climate types (Hyper Arid, Arid, Semi-Arid, Dry sub-humid, and Humid) according to the United Nations Environment Programme (UNEP) standard[42]*:* ***Hyper Arid (AI < 0.03), Arid (0.03 ≤ AI < 0.20), Semi-Arid (0.20 ≤ AI < 0.50), Dry sub-humid (0.50 ≤ AI < 0.65), and Humid (AI ≥ 0.65).” (Page 21, Line 433-438)***

Comment 6 (Results): “While population size serves as a fundamental predictor, we anticipate that its explanatory power for the variance in inequality will be modest, pointing to the critical role of other modulating factors” – Why is this anticipated and population size ruled out as a determinant of inequality? This sentence needs to be both reconsidered and justified.

Response: Thank you very much for this valuable suggestion. We agree that the original sentence was poorly phrased and could indeed be misinterpreted as dismissing population size as a determinant of inequality. We have now revised it to reflect our actual logic. Specifically, we have

revised this sentence in the Results to clearly state the rationale behind this anticipation, explicitly acknowledging population as the driver of the trend while attributing the variance to local diversity. The revised contents are detailed as follows.

*"While population size serves as the primary predictor of the global scaling trend, we anticipate that its explanatory power for the variance in inequality will be modest, given the immense cultural, economic, and morphological diversity of cities worldwide that creates significant local deviations from the universal law." **(Page 7-8, Line 134-138)***

Comment 7: One important factor to consider, is the spatial footprint of urban centres when it comes to inequalities. The more dense the cities, the less green spaces for example, and thus the more potential for inequalities in access to these. Similarly, the more dense the cities, the more likely the heat urban effect due to street canyon structures and less green spaces and trees. These considerations are overlooked in the introduction to the paper and should be given consideration to strengthen the narrative and acknowledge the caveats In the findings. They are considered in the discussion when 'population density' is referred to, but a more coherent account of the geographical dimension of inequalities is needed.

Response: Thank you very much for this valuable suggestion. We agree that the geographical dimension of inequality, particularly the role of density in shaping green space access and heat island effects, deserved more explicit attention in the Introduction. We have revised it accordingly.

The updated Introduction now includes a discussion of the trade-offs of densification, explicitly referencing green space encroachment and street canyon effects as mechanisms that link urban form to inequality. This creates a more coherent narrative that sets up the subsequent analysis. The revised contents are detailed as follows.

*"A critical but often overlooked factor is the spatial footprint of urbanization: while higher urban density drives economic agglomeration, it may inadvertently intensify local environmental stressors. For instance, densification presents significant challenges for preserving green spaces and maintaining microclimatic comfort[5], creating a "local health paradox" where the benefits of urban growth are unevenly distributed[6]." (**Page 4, Line 55-60**)*

Furthermore, we have expanded the Discussion section to further elaborate on how urban form metrics (beyond simple density) modulate these

inequalities, ensuring a coherent account of the geographical dimension throughout the manuscript. The revised contents are detailed as follows.

*"…Second, regarding urban form, we used population density as a primary proxy. Although we validated this against geometric metrics (e.g., compactness, fractal dimension) and found density to be the most robust predictor of inequality residuals (Supplementary Fig. S5), we acknowledge that density alone does not capture the full complexity of urban morphology (e.g., street canyon effects on heat trapping)." (**Page 15-16, Line 309-313**)*

Comment 8: "This finding suggests that the physical constraints of the environment, likely related to water availability for evaporative cooling, play a key role in shaping thermal disparities as cities grow" – This is a relevant findings indeed, but as noted above, there is also the likelihood that urban form (which is rather different in different climatic conditions and influences the urban structure overall) significantly impact on heat island effects. Something similar then applies to "indicating a more rapid exacerbation of green space inequity in wetter regions once a city reaches a certain size" in relation to the comments above regarding urban structure and the density of cities consideration highlighted above. I think that such 'confounding' factors must be acknowledged and be part of the discussion.

Response: Thank you very much for this valuable suggestion. We fully agree that urban form should be recognized as a potential confounding factor in the relationships we report—particularly given that urban morphology varies systematically with climate and can independently influence both heat island effects and green space inequality. In response, we have revised the Discussion and Limitations sections as described in our response to Comment 7. The revised contents are detailed as follows.

*"Although we validated this against geometric metrics (e.g., compactness, fractal dimension) and found density to be the most robust predictor of inequality residuals (**Supplementary Fig. S5**), we acknowledge that density alone does not capture the full complexity of urban morphology (e.g., street canyon effects on heat trapping)." (**Page 15-16, Line 310-313**)*

We have added this acknowledgment to the Limitations paragraph, ensuring that readers are aware of these complexities when interpreting our findings. The added contents are detailed as follows.

We thank the Reviewer for helping us clarify this important nuance.

Comment 9: "Our findings are robust to methodological choices." Which remain unexplained and unjustified on page 10. A cross-reference to the methods section here would be helpful.

Response: Thank you very much for this valuable suggestion. We fully agree that a generic reference is insufficient. We have refined the cross-reference to point specifically to the "Calculation of Inequality Metrics" subsection in the Methods, where we detail the sensitivity analysis regarding buffer sizes (300m vs. 500m vs. 800m). The revised contents are detailed as follows.

In Results section

*"Our findings are robust to methodological choices (**detailed in Methods: Calculation of Inequality Metrics**)." **(Page 11, Line 203-204)***

In Methods section

"Calculation of Inequality Metrics

To quantify intra-urban inequality, we calculated population-weighted Gini coefficients based on neighborhood-level exposure. This approach assesses the inequality of conditions experienced by the population rather than the raw spatial distribution of the variables themselves. The process involved a focal analysis centered on each 100m population grid cell. For

*each population cell, we calculated the mean green space coverage, LST, and NTL intensity within a surrounding 500m radius buffer. The population count of the central grid cell was then paired with these neighborhood-level mean values. This resulted in a series of paired (population, exposure_value) data points for each city. The Gini coefficient, a measure of statistical dispersion ranging from 0 (perfect equality) to 1 (perfect inequality), was then computed for each city and each variable. This calculation was performed in the R programming environment41 using the gini.wtd function from the dineq package, with the neighborhood-level mean values serving as the value vector and the corresponding cell populations as the weight vector. The primary results presented in this paper are based on the 500m buffer analysis, while additional calculations using 300m and 800m buffers were performed for sensitivity analysis (Supplementary Fig. S3)." (**Page 19-20, Line 385-401**)*

Comment 10: "urban form, proxied by population density, explains a significant portion of the remaining variance in inequality" – I am unconvinced that urban form can be so bluntly proxied by population density. While this holds true for definite urban cores/areas, this generalisation underestimates the inequality inherent in urban form and population density themselves with richer areas typically having lower density and more open urban forms. I understand that the paper is trying to

look at the issue from a global and strategic perspective, but these other considerations merit their acknowledgement and reflection.

Response: We thank the reviewer for this insightful comment, which touches upon a fundamental challenge in urban form quantification. We completely agree that population density is an imperfect proxy in theory, and we appreciate the opportunity to empirically test its validity against other geometric descriptors. To rigorously test whether population density is a valid proxy despite these complexities, we conducted a new sensitivity analysis. To be specific, we calculated three additional geometric metrics (Compactness, Fractal Dimension, Elongation) and Road Network Density for all cities and analyzed their correlation with inequality residuals.

The results, presented in the new Supplementary Figure S5, show that population density consistently exhibits the strongest explanatory power compared to the geometric indices. We have revised the Results section to reflect this validation as follows.

In Results section

"To validate the robustness of this proxy, we further conducted a sensitivity analysis... As shown in Supplementary Figure S5, population density consistently exhibited the strongest explanatory power for the variance in

inequality residuals across all dimensions compared to geometric forms."

(Page 11, Line 216-221)

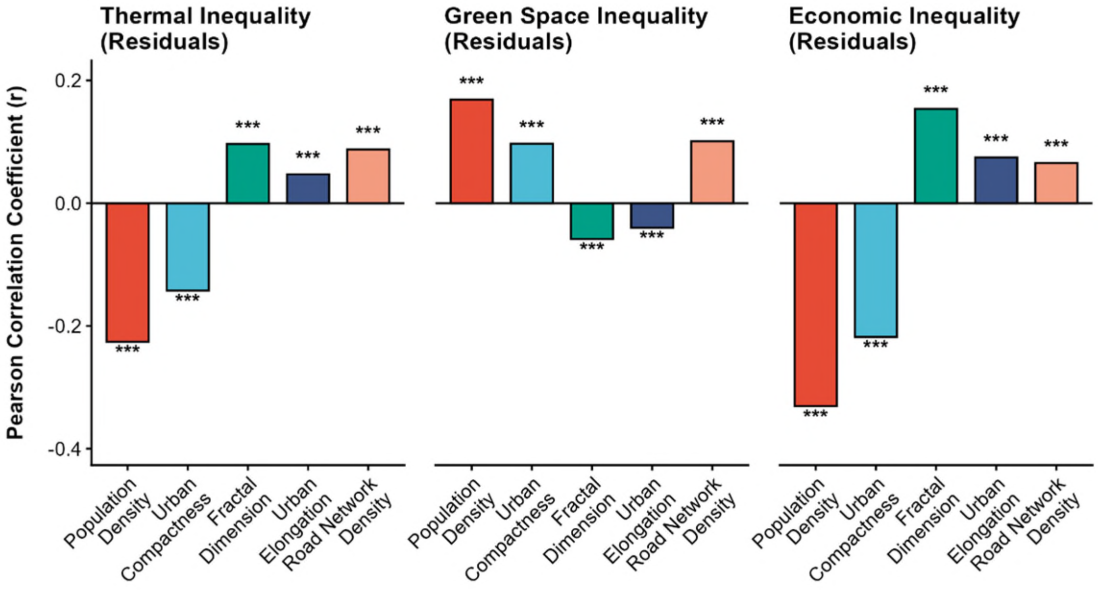

Supplementary Figure S5 | The association between urban form metrics and the residuals of inequality scaling laws. *The bar chart displays the Pearson correlation coefficients (r) between five key urban form indicators (Population Density, Urban Compactness, Fractal Dimension, Urban Elongation, and Road Network Density) and the residuals of the global scaling models for thermal, green space, and economic inequality. The residuals represent the deviation of a city's inequality from the value predicted by its population size alone. Asterisks denote statistical significance levels (*** $P < 0.001$, ** $P < 0.01$, * $P < 0.05$). The results indicate that compact and dense urban forms are generally associated with lower thermal and economic inequality (negative correlations) but higher green space inequality (positive correlations), highlighting a potential trade-off in urban planning.*

Comment 11: Figure 1 – it is not immediately clear what the conceptual schematics mean to illustrate. The right/left split is misleading as inequalities are never so defined in urban areas; these can take the form of pockets rather than such radical splits. Also, the green/yellow/orange backgrounds are not explained and add to the confusion.

Response: Thank you very much for this valuable suggestion. We agree that the original schematic was ambiguous and could be misinterpreted. To address this, we have completely redrawn the figure and thoroughly revised its caption to enhance clarity. The new caption clarifies that the figure is a schematic abstraction illustrating statistical divergence (spatial sorting), not a literal geographic map. The goal is to illustrate a conceptual point, not to map real-world inequality. The updated figure and caption are provided as follows.

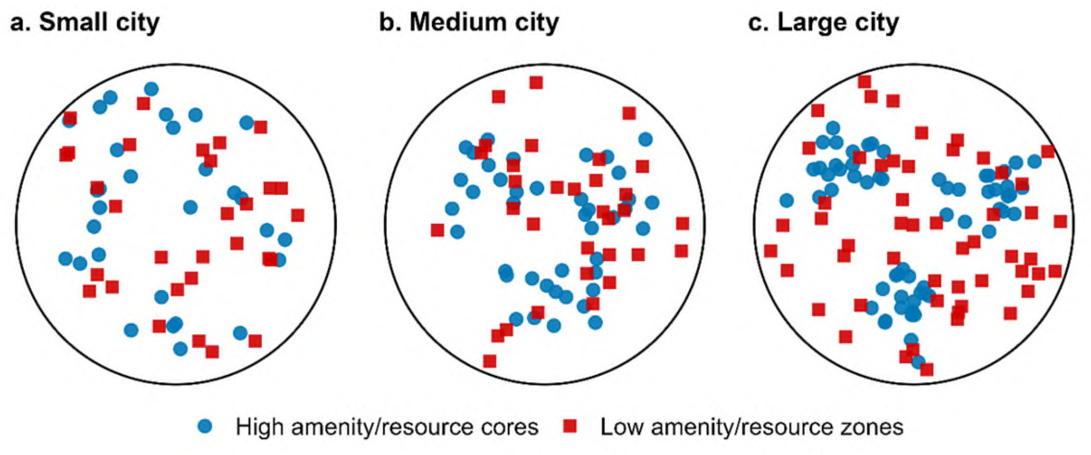

Figure 1 | Conceptual framework for the scaling of intra-urban inequality. *The schematic illustrates the hypothesis that inequality scales super-linearly with city size due to spatial sorting. In small cities (**a**), high-amenity resources (blue circles) and low-amenity zones (red squares) are relatively mixed, exhibiting a stochastic distribution. In medium-sized cities (**b**), spatial sorting is discernible, with resources showing partial aggregation. In large cities (**c**), high amenities are concentrated into distinct clusters or "pockets", which are spatially segregated from lower-amenity areas. The observation that larger cities exhibit a more clustered spatial structure compared to the mixed distribution in smaller cities underlies the disproportionate increase in inequality (Gini coefficient) relative to population size (α>0). Note: The spatial separation depicted is a schematic abstraction to illustrate the statistical divergence of inequality (widening gap between rich and poor zones) and does not represent a specific geographic layout (e.g., East-West divide).*

Comment 12 (Discussion): "predictive science capable of forecasting the evolution of inequality as a function of urban growth." Yet the degree of inequality is very context specific, and influenced and modulated by many factors which again, the paper fails to acknowledge.

The discussion is otherwise well articulated and some relevant and important points are made (e.g. "For instance, the finding that low-income nations are on a steeper trajectory of green space inequality highlights the urgent need for well-funded policies that prioritize equitable greening strategies (…)".

Response: Thank you very much for this valuable suggestion. We appreciate the Reviewer's positive assessment of the articulation and policy relevance of our Discussion. At the same time, we fully accept the Reviewer's critique regarding the complexity and context-specificity of inequality. We agree that our cross-sectional results should not be framed as deterministic forecasts of temporal evolution, and we have systematically moderated the predictive language throughout the manuscript. Specifically, we have revised the Discussion section to define our contribution as "characterizing the structural state of inequality as a function of urban scale," rather than forecasting future evolution as follows.

"On the other hand, it elevates environmental justice research from predominantly case-specific descriptions of inequity[22,24] to a predictive science capable of characterizing the structural state of inequality as a function of urban scale. " ***(Page 13, Line 250-252)***

Furthermore, to better acknowledge the influence of local contexts as requested, we have significantly expanded the Discussion section. We now explicitly warn that "…longitudinal scaling exponents may diverge from cross-sectional ones due to path dependencies…" thereby clearly differentiating individual city trajectories from the global trend. This addition complements our discussion on the model's explanatory power,

where we reinforce that the modest R^2 values highlight that "while population size provides a universal baseline... other factors, which we explore, play a critical role in modulating this process." The revised contents are detailed as follows.

*"As noted by Pumain[33], Depersin and Barthelemy[18], and Keuschnigg et al.[19], longitudinal scaling exponents may diverge from cross-sectional ones due to path dependencies." (**Page 15, Line 307-309**)*

The modest R^2 values in our global models do not diminish the significance of this finding; rather, they highlight that while population size provides a universal baseline for the scaling of inequality, other factors, which we explore, play a critical role in modulating this process.

We believe these revisions appropriately temper our claims while preserving the paper's core insights.

Comment 13: "our analysis is cross-sectional and does not capture the temporal evolution of inequality within individual cities" – the year of the data this study was based on is not acknowledged. This only comes in the methods section but acknowledging this from the outset (e.g. results

section) would strengthen the validity of the results and discussion by providing a clear and current timeframe to the reader.

Response: Thank you very much for this valuable suggestion. We fully agree that stating the timeframe early in the manuscript strengthens the validity of the results. In response, we have revised the opening sentence of the Results section to explicitly mention the year(s) of the data used in this study, providing readers with a clearer temporal context from the outset. We appreciate your insightful review and guidance.

The revised contents are detailed as follows.

*"Our analysis, encompassing over 11,000 cities worldwide (**based on data circa 2024, defined as high-density urban centers, see Methods**) (Fig. S1), reveals that intra-urban inequalities are not random but follow predictable scaling laws with city size." (**Page 7, Line 132-134**)*

Comment 14 (Limitations): The limitations paragraph of the discussion section should address some of the aspects raised above (e.g. urban footprint, urban form, etc.).

Response: Thank you very much for this valuable suggestion. We have added a comprehensive Limitations paragraph at the end of the Discussion. This paragraph explicitly addresses the cross-sectional nature of the study and the complexities of the urban footprint and form. Specifically, we acknowledge the limitations of using population density as a primary proxy and highlight that density alone does not capture the full complexity of urban morphology (e.g., explicitly referencing street canyon effects). The revised contents are detailed as follows.

"Our study has several limitations. First, our scaling analysis is based on cross-sectional data. While this approach effectively characterizes the macro-structural state of the global urban system[17], it should not be conflated with the longitudinal trajectories of individual cities. As noted by Pumain[33], Depersin and Barthelemy[18], and Keuschnigg et al.[19], longitudinal scaling exponents may diverge from cross-sectional ones due to path dependencies. Second, regarding urban form, we used population density as a primary proxy. Although we validated this against geometric metrics (e.g., compactness, fractal dimension) and found density to be the most robust predictor of inequality residuals (Supplementary Fig. S5), we acknowledge that density alone does not capture the full complexity of urban morphology (e.g., street canyon effects on heat trapping). Third, we use remote sensing-based proxies for complex phenomena; LST is not

equivalent to human thermal comfort, and Nighttime Lights capture only a facet of economic activity. Fourth, our Gini coefficient approach quantifies overall inequality but does not reveal the specific social groups (e.g., defined by race or ethnicity) who are most affected. Finally, our scaling analysis reveals strong correlations, but establishing the precise causal mechanisms requires further, more detailed investigation, potentially integrating micro-level data as has been demonstrated by Arvidsson et al.[20]*"*

(Page 15-16, Line 304-320)

Comment 15 (Methods): The methods section needs to justify why these three parameters are examined in the first place (see related comment above).

Response: Thank you very much for this valuable suggestion. As detailed in our response to Comment 3, we have addressed the justification for variable selection by adding a dedicated theoretical framework in the Introduction. We chose to centralize this motivation at the beginning of the manuscript to immediately establish the link between these parameters (LST, Green Space, and NTL) and human health/wellbeing. This ensures the rationale is clear from the outset, allowing the Methods section to focus on the technical details of data processing and metric calculation.

*“To understand these trade-offs, we focus on three critical dimensions of the urban environment that are directly linked to human health and wellbeing: thermal exposure, green infrastructure, and economic activity. First, Land Surface Temperature (LST) serves as a key indicator of heat exposure risk. The uneven distribution of urban heat often creates a "landscape of thermal inequity," where marginalized populations are disproportionately exposed to heat stress7–9. Second, Urban Green Space (UGS) acts as a vital health resource. Systematic reviews confirm that access to green space significantly reduces all-cause mortality by mitigating heat islands, encouraging physical activity, and facilitating psychological restoration10,11. Third, Nighttime Light (NTL) intensity provides a robust high-resolution proxy for the intensity of economic activity at the micro-scale12,13, reflecting the spatial distribution of economic wellbeing.” (**Page 4-5, Line 62-72**)*

Comment 16 (Methods): “The socioeconomic stratification was based on the four national income levels (High, Upper Middle, Lower Middle, and Low Income) provided by the GHS-UCDB.” Provide a reference to back this up and include an outline of income breaks behind those categories for clarity.

Response: Thank you very much for this valuable suggestion. We acknowledge that this information should have been included in the original manuscript. We have revised the Methods section to explicitly cite the World Bank classification standards and list the specific GNI per capita thresholds used for the stratification. We appreciate your thorough review in helping us improve the clarity and rigor of our methodology.

The revised contents in the "Scaling Law Analysis" in the Method section are detailed as follows.

*"The socioeconomic stratification was based on the four national income levels provided by the GHS-UCDB, which aligns with the World Bank classification (fiscal year 2024) based on 2022 GNI per capita: Low Income (<= $1,135), Lower-Middle Income ($1,136–$4,465), Upper-Middle Income ($4,466–$13,845), and High Income (> $13,845)[42]" (**Page 21, Line 429-433**)*

We have also added the corresponding citation to the reference list in the revised manuscript."

We sincerely thank you again for your constructive comments, which has greatly strengthened our work. In the submitted revision, all modified

contents are marked in yellow. We hope these revisions meet your approval. Should any further adjustments be required, we are fully prepared to make them.

Responses to the reviewers

Reviewer #1 (Remarks to the Author):

The authors took my suggestions on the first version of their article very well into account. They clarified the vocabulary without confusing cross-sectional and longitudinal analyses. The limitations of the study due to the nature of the data were also clearly stated. I believe this second version is ready for publication.

Response: Dear Reviewer #1, thank you again for your positive evaluation, your final endorsement, and all the constructive suggestions that helped shape this manuscript. We sincerely appreciate your time and effort.

Reviewer #2 (Remarks to the Author):

I am satisfied that the authors have appropriately addressed my comments by clarifying certain considerations and acknowledging important research shortcomings and confounding factors. The article reads better now and the description of the methodology and results is more comprehensive.

Response: Dear Reviewer #2, thank you again for your careful reading, your final endorsement, and the rigorous critiques that significantly improved the comprehensiveness of our work. We deeply appreciate your contribution to this research.
